# How do people perceive listeners?

**DOI:** 10.1098/rsos.241550

**Published:** 2025-04-09

**Authors:** Guy Itzchakov, Geoffrey Haddock, Sarah Smith

**Affiliations:** ^1^ University of Haifa, Haifa, Israel; ^2^ Cardiff University, Cardiff, UK

**Keywords:** listeners, reverse correlation, social perception

## Abstract

Listening is essential in shaping social interactions, relationships and communication. While listening research has generated significant insights on how speakers benefit from good listening, one fundamental question has been largely overlooked: how do people perceive listeners? This gap is crucial for understanding how perceptions of listeners impact relational dynamics. In three studies (two preregistered; total *N* = 1509), we assessed the attributes and behaviours associated with good and bad listeners, and whether the favourability of these attributes and behaviours impact downstream consequences. In Study 1, participants identified an acquaintance they judged as a good or bad listener. Good listeners were rated higher in positive listening attributes and behaviours, which mediated their perceived warmth, competence and values. Study 2 replicated this using a reverse correlation technique: one sample generated faces of a good or bad listener, which were then evaluated by a second, naïve sample. Consistent with Study 1, good listener faces were rated higher in positive listening attributes and behaviours, mediating perceptions of warmth, competence, humility and values. Study 3 extended Study 2 by showing that the effects were not due to a general positivity bias, demonstrating the significant interpersonal consequences of being perceived as a good or bad listener.

## 1. Introduction

Imagine that you are on a speed date. You are sitting across from someone who listens to you well. They give you their full attention, their eyes sparkling with interest, nodding along and asking thoughtful questions to understand you better. Then, you move to the next seat, where your conversation partner is a poor listener. They seem distracted, looking around the room and displaying expressions of boredom. Who are you more likely to ask for a real date? The answer, in this case, seems pretty straightforward.

Listening is an essential aspect of human relationships and a frequent part of our daily lives. People spend about 70–80% of their day engaged in some form of communication, with 45–55% of that time dedicated to listening, which is more than any other communicative activity [1]. In certain contexts, the time spent listening is even higher. For example, on average, workers spend approximately 55% of their work time listening, while managers spend about 63% of their working day engaged in listening [1].

To date, listening research has focused primarily on how speakers benefit from being the recipient of good listening. This literature has found that being listened to has a range of positive outcomes, such as reducing a speaker’s social anxiety [2], stress [3], loneliness [4] and attitudinal polarization [5], as well as increasing a speaker’s psychological safety [6], autonomy and relatedness [7–9]. Relatedly, poor listening hinders the speaker’s speech fluency [10], reduces the speaker’s willingness to self-disclose [8], and negatively impacts memory [11] and creativity [12].

In this paper, we ask an overarching and fundamental question: *how do people perceive and evaluate good versus bad listeners?* That is, what attributes and behaviours do people ascribe to good versus bad listeners? What are the downstream consequences of being perceived as a good or bad listener, and what underlies these evaluative consequences? How do people visually represent good versus bad listeners? Answering these questions is essential because understanding how people perceive and evaluate listeners enhances our knowledge of psychological processes fundamental to interpersonal interactions, by further elucidating the cognitive and affective mechanisms that underlie human communication.

While listening plays a fundamental role in social interactions [13,14], the implications for shaping how individuals perceive and evaluate listeners have been underexplored. To our knowledge, one piece of work [15] has directly considered how people conceptualize listeners, which we describe below. The present research builds upon this work by examining listeners within the framework of social cognition, emphasizing its broader implications for interpersonal evaluations and social relationships. Listeners do not only engage in understanding content but also in signalling attentiveness, respect and engagement to their conversation partners. These qualities contribute to how people form impressions and navigate social dynamics. These considerations are vital for advancing theories of interpersonal communication and social interaction by positioning the listener as a key factor in fostering mutual understanding [16], building social connections [4,17] and enabling effective collaboration across various contexts [18,19]. By situating the listener within this broader framework, the research seeks to highlight its importance not just as a skill but as a relational practice with profound implications for personal and professional domains.

## 2. What do we know about listeners?

Most listening research to date has focused on listening at the *behavioural* level, specifically, assessing the effects of good listening (e.g. [19,20]). This is essential for understanding social interaction processes and their outcomes [21,22]. However, another essential component of listening that has been largely neglected is how listeners are perceived at the *person* level. Specifically, what characteristics do people ascribe to good versus poor listeners, and how do these perceptions influence interpersonal dynamics? For example, individuals may be more inclined to discuss sensitive topics, such as social or political attitudes, with those they perceive as good listeners. Moreover, individuals should attribute positive social traits to good listeners, such as care, responsiveness and attentiveness. Conversely, individuals should attribute negative social traits to bad listeners, such as impatience, coldness/detachment and selfishness. Moreover, employees may share less information with managers perceived as poor listeners, particularly regarding difficulties and problems, which can hinder effective organizational functioning.

In relevant research, Bodie *et al*. [15] published a series of studies that examined this question through the lens of implicit theories. These theories, which are mental representations of people and actions, shape the impressions we form of others. Bodie *et al*. [15] identified 19 specific behaviours—both verbal (e.g. subject-appropriate responding, asking questions) and nonverbal (e.g. maintaining eye contact, using appropriate body language)—that were associated with listening competence during initial interactions. Verbal behaviours were found to be more strongly related to listening competence than non-verbal behaviours, mainly because they were linked to more attributes that are centrally relevant when people evaluate listening. Additionally, Bodie *et al*.’s work iteratively built an empirical database of the attributes (what competent listening is) and behaviours (what competent listeners do), creating an evidence-based, preliminary model for understanding the role and structure of implicit theories of listening. This model offers a foundation for investigating how these theories influence impression formation during initial interactions [15].

Recent theorizing suggests that listening is more than a set of behaviours, and there is no specific set of behaviours that universally leads to perceptions of good (or bad) listening. Rather, prominent perspectives note that evaluations of listening depend on the extent to which a listener is willing to be devoted to the speaker [23]. This highlights the need to study listening also at the person level, as it emphasizes the role of a listener’s underlying intentions and willingness to engage. Focusing on these factors can reveal how personal attributes, like warmth and humility, shape perceptions of listening quality. Further, this approach may lead to more nuanced insights into how listening varies across different contexts and relationships. Therefore, in the present research, we study both the attributes and behaviours that represent listeners. In particular, because of its ubiquity in the literature [21] and centrality to our core social relationships [17,24,25], we focus on listening in a conversation between two people (hereafter: dyadic listening).

## 3. What outcomes might be linked with being perceived as a good or bad listener?

In addition to assessing the attributes and behaviours perceived to represent good versus bad listeners, we also sought to shed insights on the downstream effects linked with being seen as a good or bad listener. Ralph Nichols, a pioneer in this field, conducted the first study on this subject in 1948. In that study, professors described students in the top and bottom tertiles of a listening comprehension test. Those in the upper tertile were characterized as ‘more attentive during classroom activities and more conscientious in their … work habits’ [26, p. 160]. Nichols also found that listening is associated with skills and habits, general intelligence, specific facets of intelligence and certain aspects of the mental set [27–29].

In our studies, we focused on warmth and competence, given their essential role in person perception [30]. We also focused on values, given their essential role in guiding people’s attitudes and behaviour [31]. Below we elucidate how they should be linked with being judged as a good or bad listener.

### 3.1. Warmth and competence

By definition, a good listener should be perceived as being both warm and competent [21]. Regarding warmth, ample evidence suggests that speakers feel socially connected with good listeners, as measured by relatedness [8,16], liking (e.g. [32–35]) and positivity resonance [4]. Together, these findings suggest that good listeners should be perceived as possessing greater warmth than bad listeners. Relatedly, a study examining the relative similarity of implicit theories of listening, communication and general social skills suggests that our assessments of conversation partners as good or bad listeners are closely related to how we evaluate them as socially skilled or unskilled individuals [36].

There is also reason to believe that a good listener will be perceived as more competent than a bad listener. Competence encompasses an individual’s ability to effectively achieve their goals and succeed in tasks. This includes attributes such as intelligence, efficacy and creativity [37]. To our knowledge, the impact of listening on perceived competence has not been directly tested. However, being a good listener is recognized as an essential leadership skill [38,39] and is important for leadership emergence, which requires competence [40]. Furthermore, studies with newly formed teams found that perceptions of a team member’s listening quality were closely linked to perceptions of their competence to lead the team [41,42]. As such, good listeners should be perceived as being more competent than bad listeners.

### 3.2. Values

Values are ideals that serve as guiding principles in a person’s life, influencing attitudes and behaviour [31,43,44]. The most influential model of values was designed by Schwartz [45]. Schwartz’s circumplex model differentiates among four core value types that fall along two dimensions. Along one dimension, s*elf-transcendence* values reflect concern for others’ welfare and include helpfulness and equality, whereas *self-enhancement* values reflect attention to personal status and include power and achievement. Along the second dimension, *openness to change* values reflect pursuing personal interests in unknown directions and include self-direction and stimulation, whereas *conservation* values reflect the preservation of the status quo and include tradition and obedience.

Regarding the self-transcendence and self-enhancement dimensions, being a good listener requires devotion to the speaker, specifically engaging in the conversation with and for them [23]. To achieve this, good listeners need to prioritize the needs of their speakers over their own. This suggests that good listeners should be perceived as attaching greater importance to self-transcendence values relative to bad listeners. Regarding self-enhancement, bad listeners try to exert control over conversations [46], asking irrelevant questions that satisfy their curiosity about the speaker’s needs [47,48], and interrupting speakers before they finish talking [49]. Together, this suggests that good listeners should be perceived as attaching less importance to self-enhancement values relative to bad listeners.

Regarding the openness to change and conservation dimension, a key dimension of good listening is undivided attention towards the speaker [21], which requires motivation to learn about them. Good listening also requires adopting a non-judgemental approach towards the speaker [21,50]. To achieve such a state individuals need to be open to listening to new points of view, including those who they might disagree with [4]. This suggests that good listeners should be perceived as attaching greater importance to openness to change values relative to bad listeners. Finally, we had no *a priori* rationale regarding why being perceived as a good versus bad listener would impact judgements on conservation values.

## 4. Integrating listener attributes, listener behaviours and outcome variables: a moderated mediation model

In addition to testing for differences in evaluations of good versus bad listeners, we explored the mechanisms through which thinking about a good versus bad listener influences the outcome variables described above. We tested a model in which we expected the effect of being perceived as a good or bad listener on our outcome variables would be mediated by the valence of the listening attributes and behaviours associated with a target, with more positive valence ratings on listening attributes and behaviours leading to more positive outcomes. Further, we tested whether any mediation would be moderated by participants’ self-perceived listening. When individuals perceive themselves as good listeners, we expect that they should be particularly likely to appreciate the benefits of good listening, leading to more positive evaluations of good listening behaviours compared with individuals who perceive themselves as bad listeners. This reasoning is consistent with research demonstrating that people place high social value on attributes that they believe they possess [51,52]. We were uncertain as to whether participants’ self-perceived listening would influence perceptions of bad listening behaviours. On the one hand, good listeners might be especially likely to denigrate behaviours they associate with bad listening. On the other hand, given the relative dissociation between constructive and destructive listening [53], complimentary effects might not be found.

**Figure 1.**
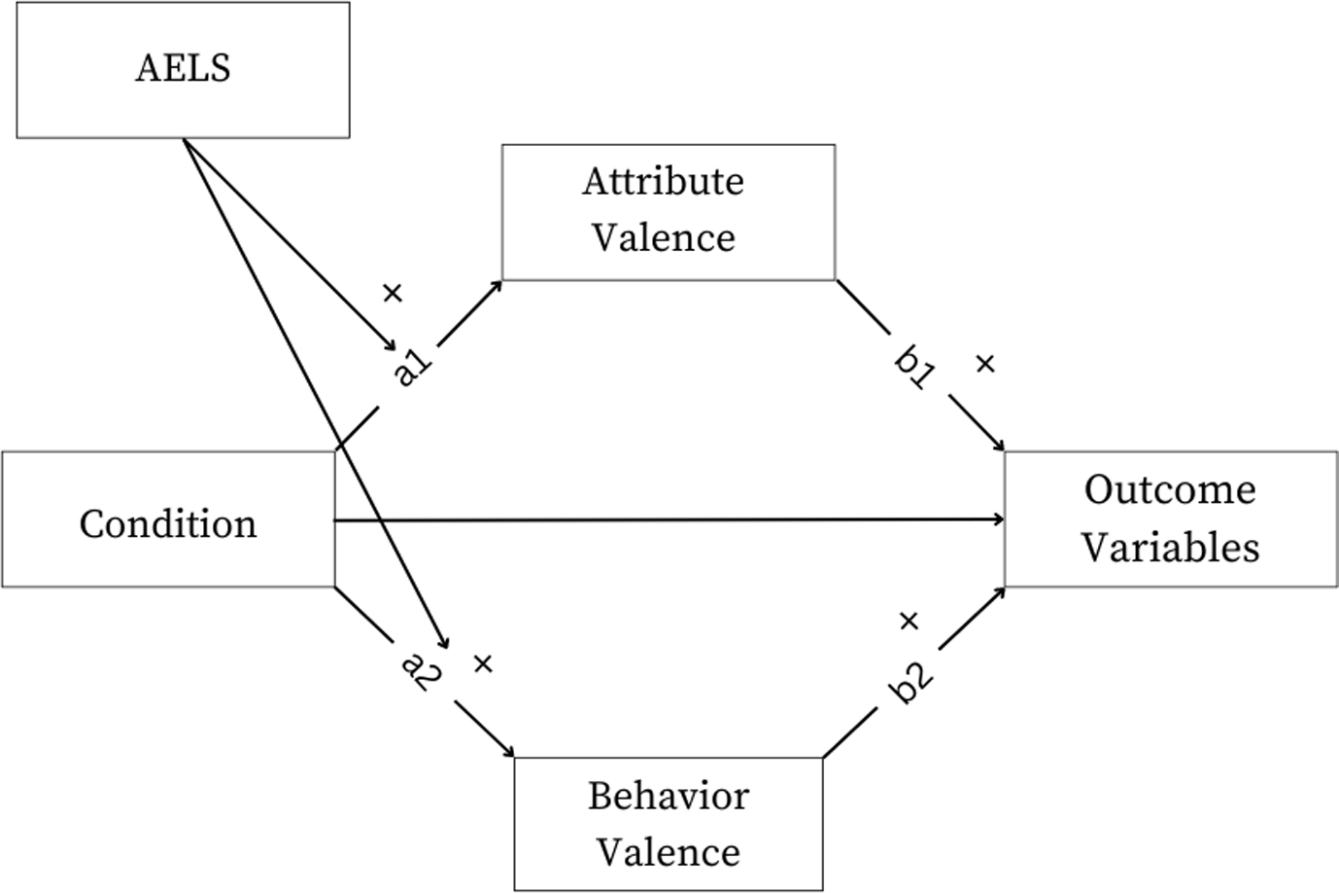
Proposed moderated mediation model.

In sum, we tested the following hypotheses (the model is outlined in figure 1):

**Hypothesis 1a**: *Good listeners will be perceived as having more positive attributes than bad listeners.*

**Hypothesis 1b**: *Good listeners will be perceived as having more positive listening behaviours than bad listeners.*

**Hypothesis 1c**: *Good listeners will be perceived as having fewer negative attributes than bad listeners.*

**Hypothesis 1d**: *Good listeners will be perceived as having fewer negative listening behaviours than bad listeners.*

**Hypothesis 2**: *Good listeners will be perceived as warmer than bad listeners.*

**Hypothesis 3**: *Good listeners will be perceived as more competent than listeners.*

**Hypothesis 4**: *Good listeners will be perceived as more humble than bad listeners.*

**Hypothesis 5**: *Good listeners will be perceived as having higher self-transcendence values than bad listeners.*

**Hypothesis 6**: *Good listeners will be perceived as having lower self-enhancement values than bad listeners.*

**Hypothesis 7**: *Good listeners will be perceived as having higher openness values than bad listeners*

**Hypothesis 8**: *The effects of the experimental condition will be mediated simultaneously via (i) positive and (ii) negative listening attributes and (iii) positive and (iv) negative listening behaviours (see* *figure 1**).*

**Hypothesis 9** *: The indirect effects of listening attributes and behaviours on the dependent variables will be moderated by participants' perceptions of their own listening qualities, such that the better participants perceive their own listening qualities, the stronger each indirect effect will be.*

## 5. Overview of studies

We report three studies addressing our fundamental question. In Study 1, participants thought about someone they knew whom they felt was a good (or bad) listener. They reported the attributes and behaviours that made this person a good or bad listener, and they evaluated them on their perceived values, warmth and competence. We tested whether good listeners were associated with different (and more positive) listening attributes and behaviours compared with bad listeners and whether such differences would mediate effects on judgements of the target’s perceived values, warmth and competence, potentially moderated by participants’ self-perceived listening abilities.

In our pre-registered Study 2, we were interested in how people visually represent good or bad listeners—that is, what people think a good or bad listener looks like. Using a reverse correlation procedure [54], we had one sample of participants generate a classification image of a good or bad listener. These classification images were then evaluated by a separate sample, who were given no information about the images or how they were generated. We tested whether these participants would see the good and bad listener faces as (i) possessing positive and negative listening attributes and (ii) engaging in positive and negative listening behaviours. We also tested whether these naïve raters would judge the good versus bad listening faces as differing in their perceived warmth, competence and humility, and holding different values, while also testing for moderated mediation.

Finally, in our pre-registered Study 3, we sought to replicate Study 2 and extend it by considering whether attributes associated with good or bad listeners are applied to other facial images.

Several themes were consistent across all of our studies. First, we used a bottom-up approach, where participants described their own personal views of what makes someone a good versus bad listener, and how they visually represent good versus bad listeners. Second, we independently assessed the perceptions of good listeners *and* bad listeners. This is because research has demonstrated that good (i.e. constructive) listening and bad (i.e. destructive) listening are best conceptualized as separate dimensions, rather than endpoints along a single continuum [21,53]. All of our studies used non-student samples, to obtain a more diverse representation of how people perceive and evaluate good and bad listeners. The research received an IRB # EC.23.04.25.6791G. All studies, measures, manipulations and participant exclusions are reported in the manuscript.

In addition, we wish to note that we conducted a study that assessed how people perceive good and bad *listening*. Given that the focus of the present manuscript is on the perception of *listeners* and we did not measure any of the dependent variables in this extra study, we decided not to include this study in the main text, to enhance the paper’s conceptual coherence. However, this study (labelled Pilot Study), including its results, is described in detail in the electronic supplemental materials and the data and syntax can be found on the project’s OSF page.

### 5.1. Open research practices

This manuscript adheres to accepted transparency and openness guidelines [55]. The data, codes and preregistrations (Studies 2 and 3) are available at this OSF link: https://osf.io/rz8p6/?view_only=a519d77551d24e49ae78f571aa15579a

## 6. Study 1

### 6.1. Method

#### 6.1.1. Participants

A total of 381 participants (*M*_age_ = 37.6 years; s.d. = 12.8; 57.4% identified as female, 40.5% as male, 1.8% as other, 0.3% preferred not to say; 63% with a bachelor’s degree or higher) were recruited via Prolific. Participants were paid £1.25 for their participation. Sensitivity analysis indicated that the smallest effect size that this sample can detect with a power of 80% and *α* = .05 is Cohen’s *d* = 0.29 [56].

#### 6.1.2. Procedure

Participants were randomly assigned to think of someone they knew who they considered to be a good or bad listener in a conversation between two people. After selecting their target, participants reported both the listening attributes and behaviours that make their target a good (or bad) listener, with these attributes and behaviours rated for valence. Next, participants rated their target on the degree to which they were warm and competent, as well as indicating their perception of the target’s values. After completing these measures, participants rated their own listening alongside some questions not pertinent to the paper.

#### 6.1.3. Measures

#### Listening attributes and behaviours

First, participants provided the person’s name before listing (i) five attributes describing the selected target they considered as a good/bad listener and (ii) five behaviours they felt made the person a good/bad listener (these tasks were presented in random order). Participants were given five text boxes for each task, with each text box limited to 50 characters. Participants were instructed to generate their responses independently, without using any online tools. Next, participants rated the valence of each word they had reported on a scale from 1 (extremely negative) to 7 (extremely positive). Attribute and behaviour scores for each participant were calculated by averaging these valence ratings.

#### Warmth and competence

Warmth was assessed by averaging responses to two questions on a 100-point sliding scale (with endpoints of not at all and extremely). The two questions asked participants to report the degree to which their target is (i) warm and (ii) likable, *r* (367) = .65, *p* < .001. Competence was measured by averaging the responses to two questions scaled along the same 100-point scale used to measure warmth. The two questions asked participants to report the degree to which their target is (i) competent and (ii) successful, *r* (367) = .53, *p* < .001. This approach aligns with the assessment of warmth and competence in other research (e.g. [57,58]).

#### Values

Participants completed a brief version of the Schwartz Values Survey, where they reported the extent to which their target would perceive Schwartz’s four core value types as personally important (59). One item represented each of self-transcendence (e.g. honesty, equality, forgiveness, protecting the environment), self-enhancement (e.g. ambition, wealth, power, success), openness (e.g. freedom, curiosity, adventurousness, excitement) and conservation (e.g. politeness, respect for tradition, obedience, social order) value types, with responses provided on a 100-point sliding scale (with endpoints of not at all and a great deal).

#### Self-perceived listening

To measure participants’ perceptions of their own listening behaviour, they completed the Active-Empathic Listening Scale (AELS [60]). A sample item of this scale is ‘I show others that I am listening by using verbal acknowledgments’ (1 = *never or almost never true*; 7 = *always or almost alway*s *true*; *α* = .87).^1^

#### Additional measures

Participants also reported how close they were to the target (0 = *not at all close*; 100 = *extremely close*), how well they knew the target (0 = *not at all well*; 100 = *extremely well*), as well as the target’s age and gender. For exploratory purposes, participants evaluated their target on their self-esteem and standing on the Big 5 attributes. The raw data are available on the OSF link.

#### Demographics

Participants finished the study by reporting their age, gender and education level.

### 6.2. Results

#### 6.2.1. Characteristics of the selected listener

We examined the characteristics of the target selected by each participant, and whether they differed as a function of condition. These data are presented in the top section of table 1. Starting with age, there was no difference in listener age across the two conditions. Participants reported more knowledge of and feeling closer to a selected good listener relative to a selected bad listener. There was also an effect on gender. Among participants who thought of a good listener, 62.8% thought of a female target, and 36.0% thought of a male target (1.2% did not say). Among participants who thought of a bad listener, 47.9% thought of a female target, 51.5% thought of a male target (0.6% did not say). A chi-square analysis focusing on female and male responses revealed a significant difference across the good and bad listener conditions, *χ*^2^ (1) = 8.02, *p* = .005. Further analysis revealed that this effect was not moderated by participant gender (*p* = .316).

**Table 1.** Characteristics and evaluations of good and bad listeners—Study 1.

	good listener	bad listener			
	*M* (s.d.)	*M* (s.d.)	*t*	Cohen’s *d*	*p*
age	41.39 (15.12)	43.52 (17.03)	−1.22	−0.13	.225
how well known?	84.75 (18.82)	78.06 (21.68)	3.16	0.33	.002
how close?	82.44 (20.79)	63.71 (29.83)	7.01	0.73	<.001
listening attributes	6.54 (0.59)	3.25 (1.56)	26.95	2.82	<.001
listening behaviours	6.50 (0.62)	2.25 (0.81)	57.30	5.92	<.001
warm	84.87 (12.76)	49.30 (21.13)	19.51	2.04	<.001
competent	79.84 (14.37)	55.07 (21.40)	13.02	1.36	<.001
self-transcendence	81.20 (15.75)	51.11 (26.66)	13.23	1.38	<.001
self-enhancement	40.69 (24.90)	59.05 (28.52)	−6.63	−0.69	<.001
openness	67.13 (22.53)	50.33 (26.28)	6.61	0.69	<.001
conservation	63.43 (26.06)	43.91 (25.49)	7.33	0.76	<.001

#### 6.2.3. Descriptions and evaluations of listening attributes and behaviours

**Figure 2.**
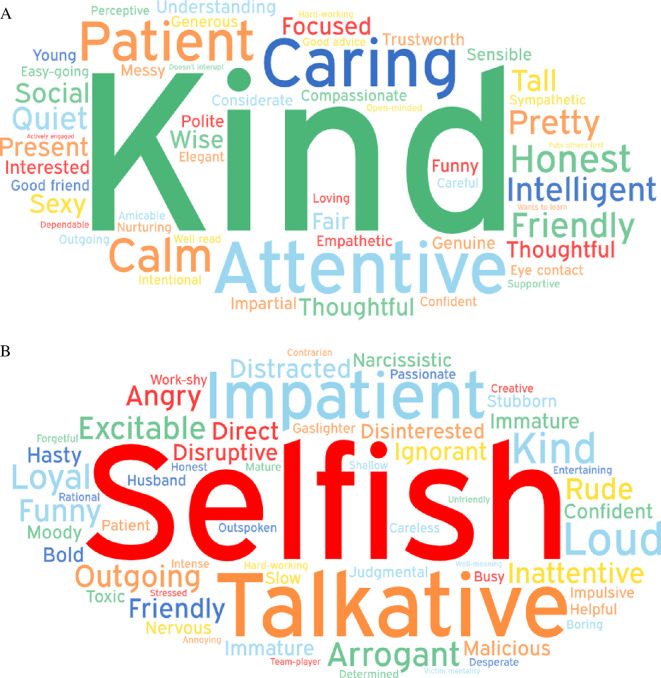
Word clouds for good (A) and bad (B) listener attributes.

**Figure 3.**
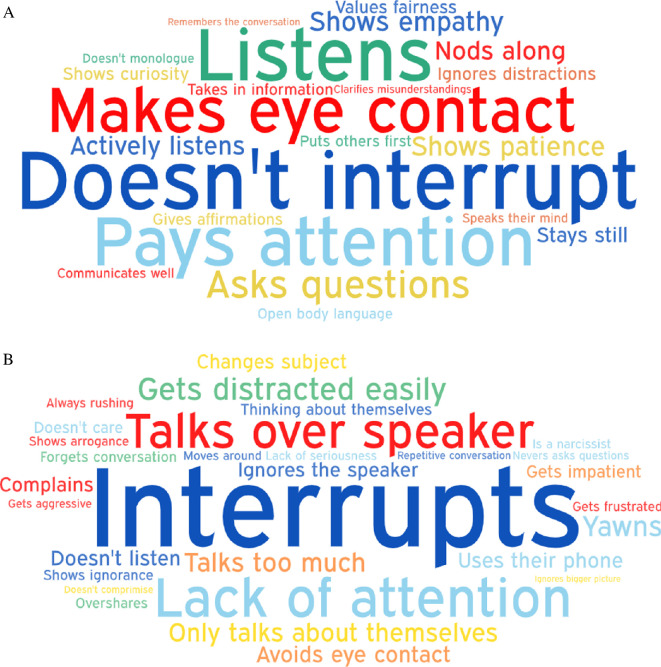
Word clouds for good (A) and bad (B) listener behaviours.

Participants used a range of attributes and behaviours to describe individuals who were good versus bad listeners. The most common attributes associated with good and bad listeners are presented in figure 2A,B, with the most common behaviours presented in figure 3A,B.

For each participant, we averaged the valence ratings of their listed attributes and behaviours. As predicted, good listeners were allocated more positive listening attributes and behaviours compared with bad listeners (both *ps* < .001, both *d*s ≥ 2.80; see table 2).

**Table 2.** Content analysis results for good and bad listeners of Study 1.

category	percentage (good listeners)	percentage (bad listeners)
kindness	30.12%	—
calmness	23.49%	—
intelligence	16.78%	—
friendliness	13.42%	—
trustworthiness	10.07%	—
positive affect	6.71%	—
disruptiveness	—	33.33%
selfishness	—	26.67%
inattentiveness	—	20.0%
anger	—	13.33%
arrogance	—	6.67%

#### 6.2.3. Differences in perceptions of good versus bad listeners

The good listener was judged to be both warmer and more competent than the bad listener (both *p*s < .001, both *d*s > 1.30). Regarding values, the good listener was judged as placing more importance on self-transcendence and openness values compared with the bad listener, with self-enhancement values showing the opposite effect (all *ps* < .001, all *ds* > |0.65|). Exploratory analysis indicated that the good listener was also judged as placing greater importance on conservation values (*p* < .001, *d* > 0.75).

#### 6.2.4. Content analysis

To supplement the word clouds we conducted a systematic content analysis. This approach allowed us to categorize participants' open-ended responses into meaningful themes, ensuring a more rigorous assessment of how listeners are perceived. Following established methodologies [61,62], the analysis involved coding responses into predefined categories based on thematic similarities. For example, words like ‘kind’, ‘supportive’ and ‘empathetic’ were grouped under ‘kindness’, while terms such as ‘distracted’ and ‘preoccupied’ were categorized under ‘inattentiveness.’ Participants' open-ended responses for attributes and behaviours were combined to create the categories for the content analysis.

As can be seen in table 2, the results of this analysis revealed distinct patterns in how participants characterize good and bad listeners. For good listeners, the most frequently mentioned attributes fell into the categories of kindness (30.12%), calmness (23.49%) and intelligence (16.78%), underscoring the importance of warmth, attentiveness and competence in effective listening. Conversely, bad listeners were most commonly described as disruptive (33.33%), selfish (26.67%) and inattentive (20.00%). These findings highlight the centrality of both behavioural and interpersonal dynamics in shaping perceptions of listening quality. By distinguishing between these attributes, the content analysis adds depth to the word cloud visualization, providing a structured and theoretically grounded framework for understanding participants’ perceptions of listeners (see table 2). The R code for the content analysis is available on the project’s OSF page (https://osf.io/rz8p6/).

#### 6.2.5. Moderated mediation analyses

To examine whether condition impacted outcomes via attribute valence and behaviour valence, with a moderating role of participants’ perceived listening abilities (hereafter: AELS), we ran a series of moderated mediation analyses using Process Model 7 [63]. All test statistics and confidence intervals are presented in table 3. We start by describing the effects of condition, AELS, and their interaction on attribute valence and behaviour valence, which are the same across all outcome variables, before discussing the effects on each outcome variable.

**Table 3.** Moderated mediation analyses for Study 1.

MEDIATOR VARIABLE REGRESSION MODELS									
	attributes			behaviours					
	*b*	*t*	CI	*b*	*t*	CI			
condition	3.31^c^	27.2	[3.07, 3.55]	4.23^c^	57.52	[4.09, 4.37]			
AELS	−0.31^b^	−2.53	[−0.56, −0.07]	−0.21^b^	−2.77	[−0.35, −0.06]			
cond * AELS	0.57^c^	3.33	[0.23, 0.91]	0.52^c^	5.03	[0.32, 0.73]			
OUTCOME VARIABLE REGRESSION MODELS									
	warmth			competence			self-transcendence	
	b	t	CI	b	t	CI	b	t	CI
direct effects									
attributes	5.93^c^	7.99	[4.47, 7.39]	6.30^c^	8.27	[4.80, 7.79]	6.07^c^	6.18	[4.14, 8.00]
behaviours	2.19	1.82	[−0.17, 4.55]	3.44^b^	2.79	[1.02, 5.86]	−0.31	−0.19	[−3.44, 2.81]
condition	6.88	1.36	[−3.10, 16.86]	−10.41^a^	-2	[−20.64, −0.18]	11.41	1.7	[−1.79, 24.62]
indirect effects									
Cond→Attr→DV	19.6		[14.01, 25.29]	20.83		[16.04, 26.41]	20.06		[12.74. 27.84]
Cond→Beh→DV	9.26		[−1.53, 19.98]	14.55		[3.76, 23.93]	−1.33		[−15.97, 13.05]
moderated mediation effects									
Cond→Attr→DV	3.38		[1.10, 5.85]	3.59		[1.20, 6.20]	3.46		[1.15, 6.23]
Cond→Beh→DV	1.14		[−0.13, 2.96]	1.79		[0.33, 3.68]	−0.16		[−1.78, 1.85]
	self-enhancement			openness			conservation		
	*b*	*t*	CI	*b*	*t*	CI	*b*	*t*	CI
direct effects									
attributes	−1.34	−1.05	[−3.85, 1.17]	1.99	1.77	[−0.22, 4.20]	2.9^a^	2.38	[0.50, 5.32]
behaviours	0.00	0.00	[−4.06, 4.07]	6.44^c^	3.54	[2.86, 10.02]	0.73	0.37	[−3.17, 4.62]
condition	−14.02	−1.61	[−31.18, 3.14]	−16.98^a^	−2.21	[−32.09, −1.86]	6.73	0.8	[−9.72, 23.19]
indirect effects									
Cond→Attr→DV	−4.43		[−12.88, 4.53]	6.57		[−0.92, 14.51]	9.63		[1.21, 17.81]
Cond→Beh→DV	0.02		[−17.86, 14.79]	27.24		[14.29, 41.46]	3.07		[-`5.40, 19.10]
moderated- mediated effects									
Cond→Attr→DV	−0.76		[−2.52, 0.80]	1.13		[−0.16, 2.79]	1.66		[0.15, 3.52]
Cond→Beh→DV	0.00		[−2.36, 1.70]	3.36		[1.22, 6.05]	0.38		[−1.78, 2.82]

^a^ *p* < 0.05

^b^ *p* < 0.01

^c^ *p* < 0.001

#### *Effects of condition and AELS on attributes and listening behaviours (paths a*_*1*_ *and a*_*2*_*)*

First, for the a_1_ path, there were significant effects of both condition (*p* < .001) and AELS (*p* = .012). As predicted, AELS moderated the association between condition and attribute valence (*p* < .001). The effect of the condition on attribute valence was greater among individuals with high AELS scores (*b* = 3.71, S.E. = 0.17, *t* = 21.51, *p* < .001, 95% CI [3.37, 4.05]) compared with individuals with low AELS scores (*b* = 2.90, S.E. = 0.17, *t* = 16.90, *p* < .001, 95% CI [2.56, 3.24]).

Similarly, for the a_2_ path, there were significant effects for both condition (*p* < .001) and AELS (*p* = .006). Again, as predicted, AELS moderated the association between condition and behaviour valence (*p* < .001). Specifically, the effect of condition on behaviour valence was greater among individuals with high AELS scores (*b* = 4.60, S.E. = 0.10, *t* = 44.07, *p* < .001, 95% CI [4.40, 4.81]) compared with individuals with low AELS scores (*b* = 3.86, S.E. = 0.10, *t* = 37.17, *p* < .001, 95% CI [3.65, 4.06]).

#### Warmth

The b_1_ path from *attribute* valence to warmth was significant (*p* < .001), such that more positive attribute scores were associated with greater perceived warmth. The b_2_ path from *behaviour* valence to warmth was non-significant, nor was the direct effect from condition to warmth.

Regarding indirect effects, the effect of condition on warmth via *attribute* valence was significant (*b* = 19.60, S.E. = 2.77, 95% CI [14.24, 25.17]), as was the effect’s index of moderated mediation = 3.38, S.E.= 1.19, 95% CI [1.10, 5.85]. The conditional indirect effect was greater among individuals with high AELS scores (*b* = 22.01, S.E. = 3.20, 95% CI [15.73, 28.37]) compared with individuals with low AELS scores (*b* = 17.19, S.E. = 2.67, 95% CI [12.07, 22.71]). The indirect effect of condition on warmth via *behaviour* valence was non-significant.

#### Competence

The b_1_ path from *attribute* valence to competence was significant (*p* < .001), as was the b_2_ path from *behaviour* valence to competence (*p* = .006). More positive attribute and behaviour valence scores were associated with greater perceived competence. The direct effect from condition to competence was also significant, (*p* = .046). However, the sign for this latter effect is opposite to that of the mean difference displayed in table 1.

Regarding indirect effects, the effect of condition on competence via *attribute* valence was significant, *b* = 20.82, S.E. = 2.68, 95% CI [16.04, 26.41], as was the effect’s index of moderated mediation = 3.59, S.E. = 1.26, 95% CI [1.20, 6.20]. The conditional indirect effect was greater among individuals with high AELS scores, *b* = 23.38, S.E. = 3.08, 95% CI [17.94, 29.81], compared with individuals with low AELS scores, *b* = 18.27, S.E .= 2.55, 95% CI [13.78, 23.70]. Further, the indirect effect of condition on competence via *behaviour* valence was significant, *b* = 14.55, S.E. = 5.19, 95% CI [3.76, 23.92], as was the effect’s index of moderated mediation = 1.79, S.E. = 0.88, 95% CI [0.33, 3.68]. Specifically, the conditional indirect effect was greater among individuals with high AELS scores, *b* = 15.82, S.E. = 5.75, 95% CI [4.05, 26.37], compared with individuals with low AELS scores, *b* = 13.27, S.E. = 4.65, 95% CI [3.48, 21.61].

#### Self-transcendence

The b_1_ path from *attribute* valence to self-transcendence was significant (*p* < .001), such that more positive attribute scores were associated with perceiving the target as attaching greater importance to self-transcendence values. The b_2_ path from *behaviour* valence to self-transcendence was non-significant (*p* = .844), as was the direct effect from condition to self-transcendence values (*p* = .090).

Regarding indirect effects, the effect of condition on self-transcendence values via *attribute* valence was significant, *b* = 20.06, S.E. = 3.85, 95% CI [12.74, 27.84], as was the effect’s index of moderated mediation = 3.46, S.E. = 1.29, 95% CI [1.15, 6.23]. This conditional effect was greater among individuals with high AELS scores, *b* = 22.53, S.E. = 4.36, 95% CI [14.36, 31.43], compared with individuals low in AELS scores, *b* = 17.60, S.E. = 3.51, 95% CI [10.99, 24.78]. The indirect effect of condition on warmth via *behaviour* valence was not significant.

#### Self-enhancement

The b_1_ path from *attribute* valence to self-enhancement (*p* = .295) and the b_2_ path from *behaviour* valence to self-enhancement were non-significant (*p* = .998). The direct effect of condition on self-enhancement was non-significant (*p* = .109).

Regarding indirect effects, the effects of condition on self-enhancement values via *attribute* valence and *behaviour* valence were both non-significant.

#### Openness

The b_1_ path from *attribute* valence to openness was non-significant (*p* = .078). The b_2_ path from *behaviour* valence to openness was significant (*p* < .001), such that more positive behaviour scores were associated with perceiving the target as attaching greater importance to openness values. The direct effect of condition on openness was significant (*p* = .028). The sign for this latter effect is opposite to that of the mean difference displayed in table 1.

Regarding indirect effects, the effect of condition on openness values via *attribute* valence was non-significant. The effect of condition on openness values via *behaviour* valence was significant, *b* = 27.23, S.E. = 6.90, 95% CI [14.29, 41.46], as was this effect’s index of moderated mediation = 3.36, S.E. = 1.24, 95% CI [1.22, 6.05]. This conditional effect was greater among individuals with high AELS scores, *b* = .29.63, S.E. = 7.59, 95% CI [15.43, 45.06] compared with individuals low in AELS scores, *b* = 24.85, S.E .= 6.25, 95% CI [13.07, 37.73].

#### Conservation

We conducted a moderated mediation analysis on conservation as an exploratory analysis. The b_1_ path from *attribute* valence to conservation was significant (*p* = .018). Both the b_2_ path from *behaviour* valence to conservation (*p* = .715) and the direct effect from condition to conservation were non-significant (*p* = .422).

Regarding indirect effects, the effect of condition on conservation values via *attribute* valence was significant, *b* = 9.63, S.E. = 4.16, 95% CI [1.21, 17.81], as was this effect’s index of moderated mediation = 1.66, S.E. = 0.85, 95% CI [0.15, 3.52]. This conditional effect was greater among individuals with high AELS scores, *b* = 10.81, S.E. = 4.62, 95% CI [1.35, 19.79] compared with individuals with low AELS scores (*b* = 8.45, S.E .= 3.74, 95% CI [1.07, 16.03]). The indirect effect of condition on conservation values via *behaviour* valence was non-significant.

### 6.3. Correcting for multiple comparisons

The Benjamini–Hochberg procedure (B–H [64]) was employed in Study 1 to correct for multiple comparisons. Unlike the Bonferroni correction, which is highly conservative and may reduce statistical power by inflating the risk of type II errors, the B–H method is designed to control the false discovery rate [65,66]. This makes it particularly appropriate for studies with numerous statistical tests, such as the present one, as it balances the need to detect true effects while minimizing false positives. Given the number and variety of tests conducted, the B–H procedure was chosen to maintain the integrity of the findings without unduly sacrificing power.

In total, 48 statistical tests were included in the analysis, encompassing *t*-tests, main effects, indirect effects and moderated mediation effects. The original *p*-values ranged from .001 ≤ *p* ≤ .998, and the range of significant tests was .001 ≤ *p* ≤ .015. After applying the B–H correction, adjusted *p*-values were calculated for each test to ensure the false discovery rate was controlled at a 5% threshold. Importantly, all significant tests (original *p*-values < .05) remained significant after correction, with adjusted *p*-values for these tests ranging from .001 ≤ *p* ≤ .045. No previously significant test became non-significant after correction.

### 6.4. Discussion

Study 1 used a bottom-up approach to examine the listening attributes and behaviours that people associated with a known acquaintance whom they perceived to be a good or bad listener. We measured downstream effects expected to be associated with being perceived as a good listener, focusing on warmth, competence and values. We also tested a moderated mediation model in which the valence of listening attributes and behaviours were expected to predict ratings of the target’s warmth, competence and values, with moderation by participants’ self-reported listening.

Our procedure shares some components with the one used by Bodie *et al*. [15] who instructed participants to engage in a retroactive imagined interaction. Participants reflected on how they introduced themselves, the topics likely discussed, and how the conversation concluded. Like our study, Bodie *et al*.’s participants were asked to imagine the listener (named ‘Alex’) as a ‘communicatively competent’ individual and list up to 20 characteristics or behaviours they believed contributed to this impression. Differently from Bodie *et al*. [15], we also assessed the attributes and behaviours of a poor listener, as well as the personality traits (i.e. warmth and competence) and values associated with listeners.

Overall, the results were consistent with our hypotheses. As predicted, people allocated more positive attributes and listening behaviours to good listeners compared with bad listeners (Hypotheses 1a to 1d). Good listeners were judged as warmer and more competent relative to bad listeners (Hypotheses 2 and 3). Good and bad listeners also differed in their perceived values, with good listeners seen as allocating greater importance to self-transcendence and openness values, and less importance to self-enhancement values, relative to bad listeners (Hypotheses 5 to 7). These latter effects are novel, as they represent the initial application of values to the study of listening.

Turning to the moderated mediation model, we found that good listeners were ascribed more positive attributes and behaviours relative to bad listeners, and the valence of the listening attributes and behaviours largely predicted warmth, competence and values. Further, on all of our outcome measures, aside from self-enhancement values, there were significant indirect effects of attribute valence and/or behaviour valence that were dependent upon AELS scores, in the expected direction. These results offer support for Hypotheses 8 and 9. Together, these results provide initial evidence highlighting the downstream consequences of being perceived by others as a good or bad listener, and moderating and mediating influences underlying these effects.

This study asked participants to think about someone they knew who they thought of as a good or bad listener. We used this approach given its alignment with our desire to examine the effects of good listening at a bottom-up level—with participants selecting their own target and freely ascribed listening attributes and behaviours to their target. While this approach offers valuable insights regarding how people think about good versus bad listeners in their everyday lives, it is important to supplement this approach with other methods. Towards that end, Study 2 used a bottom-up, *indirect* method to assess how people represent and evaluate good or bad listeners that they do not know.

Our starting point for Study 2 was to understand how people visually represent good versus bad listeners—that is, what people think good and bad listeners *look like*. We tested whether participants have different mental images of good versus bad listeners and whether other naïve participants, when shown consensual mental representations of the faces of good and bad listeners, would differentially attribute positive and negative listening attributes and behaviours to these images. Building upon Study 1, we tested whether these good and bad listening faces would be perceived as differing in warmth and competence (along with humility) and their values. Differences using this more indirect approach would speak to fundamental processes related to how people conceptualize good versus bad listeners, and provide more nuanced evidence about the consequences linked with being perceived as a good versus bad listener.

## 7. Study 2

The goals of Study 2 were twofold. First, we aimed to conceptually replicate the findings of Study 1 with the reverse correlation task [54]. This task involves two stages. First, participants in one sample generate their own mental representation of a group member, in our case a good (or bad, depending upon condition) listener. These individual representations are then averaged across generators within each condition, in our case resulting in one classification image of a good listener and another classification image of a bad listener. In the second phase, these classification images are evaluated by another sample of participants, who are unaware of how the images were generated. This task has been used to assess the impacts of mental representations of various social categories (e.g. [57,67,68]). It offers an indirect method of assessing social perception, as the classification images offer a relatively unfiltered measure of how people conceptualize social categories, with evaluations being made in the absence of any identifying information about the group.

Second, we included an additional outcome variable, humility. We included humility given its associations with greater tolerance of those with opposing views and engaged cooperation with others [69–71]. As applied to listening, research has found that good listeners are perceived as more humble by their speakers [72]. As in Study 1, participants reported their own listening ability, as we were interested in assessing the effects of self-reported listening on measured variables. Building upon Lehmann *et al*.'s [72] findings that better listeners are judged by speakers as more humble, we expect that simply being perceived as a good listener leads to being ascribed greater humility, in the absence of an actual conversation (Hypothesis 4). This study was pre-registered (https://aspredicted.org/8TZ_2VN).

### 7.1. Method

#### 7.1.1. Image generation phase

#### Participants

A total of 199 participants (*M*_age_ = 34.95 years; s.d. = 12.99; 36.7% identified as female, 61.3% as male, 1.0% as other, 1.0% preferred not to say; 66% with a bachelor’s degree or higher) were recruited via Prolific. Participants were paid £2.69 for their participation.

#### Material and procedure

The generation task was conducted using PsychoPy. Participants were randomly assigned to the good or bad listener condition. The task consisted of 410 trials, 10 of which were attention checks (see below). On each of the 400 primary task trials, participants were shown two facial images; one image was a base face superimposed with a random noise pattern, and the second image was the same base face superimposed with the opposite random noise pattern. The random noise was generated and added using the rcicr package in *R* [73]. The base face was taken from Smith *et al.* (74). Before starting the task, participants were given the following information (with ‘good’ or ‘bad’ adapted to condition):

*We are going to show you a number of pairs of faces.*

*We would like you to select which of the two faces you would consider a GOOD/BAD LISTENER in a conversation between two people.*

*So, as you decide which faces to select, think about which face best represents a GOOD/BAD LISTENER IN A CONVERSATION BETWEEN TWO PEOPLE.*

For each image pair, participants were asked:

*Which face best represents a GOOD/BAD listener?*

Each trial used the same base face with every trial including different white noise patterns. In the 10 attention check trials, a child face and an adult face were presented, and participants were asked to select the adult face. Using a criterion from previous research that participants pass at least 50% of attention check trials (see [57]), we found that all participants met that threshold.

After completing the face generation task, participants completed the AELS and the Constructive Listening Scale [53]. Including these measures allows for future research to explore how individuals high versus low on these constructs visually represent good and bad listeners.

#### Image processing

**Figure 4.**
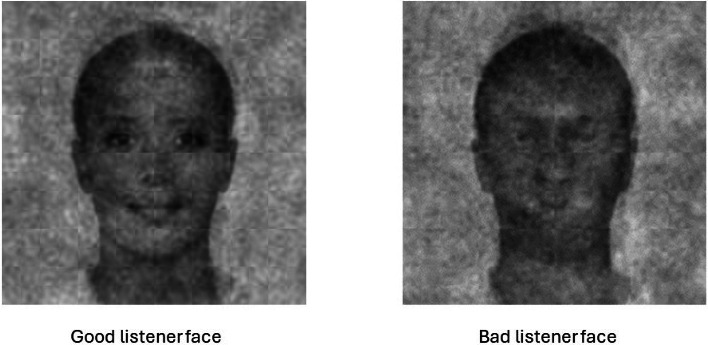
Average classification images of good and bad listeners.

Good and bad listening classification images were created using the *rcicr* package [73]. The images are presented in figure 4. These stimuli represent *condition-level* classification images. While research has suggested that condition-level classification images can inflate type I error rates [75], numerous studies have demonstrated that effects obtained using condition-level classification images are replicated when using subgroup-level classification images (e.g. [76–79]).

#### 7.1.2. Image rating phase

#### Participants

We recruited 387 participants via Prolific. Two participants were excluded for failing an attention check (see below), leaving 385 participants for analysis (*M*_age_ = 41.37 years; s.d. = 14.10; 63.1% identified as female, 35.8% as male, 0.5% as other, 0.5% preferred not to say; 61% with a bachelor’s degree or higher).^2^ Participants were paid £0.80 for their participation. Sensitivity analysis indicated that the smallest effect size that this sample can detect with a power of 80% and *α* = .05 is Cohen’s *d* = 0.29 [56].

#### Procedure

Materials were presented via Qualtrics. After providing consent, participants were told that they would be making judgements about a visually distorted image. Participants were randomly assigned to the good or bad listener condition.

#### 7.1.3. Materials

#### Listener attributes and behaviours

First, participants evaluated the assigned face on the extent to which they thought 12 attributes (six positive: attentive, caring, friendly, intelligent, kind, patient; six negative: distracted, impatient, loud, self-centred; selfish, talkative) and 12 behaviours (six positive: asks questions to the speaker, does not interrupt the speaker, makes eye contact with the speaker, pays attention to the speaker, shows empathy towards the speaker, shows patience towards the speaker; six negative: avoids eye contact with the speaker, does not pay attention to the speaker, gets distracted easily, interrupts the speaker, only talks about themselves, talks over the speaker) characterized the target as a listener during a conversation between two people. These ratings were made on a seven-point scale (1 = not at all characteristic; 7 = very much characteristic). The selected listening attributes and behaviours were among those listed most frequently by participants in Study 1. We used responses to these items to compute four indices: positive listening attributes, negative listening attributes, positive listening behaviours and negative listening behaviours. Each index showed high reliability across the good listener face and bad listener face conditions (all *α* > .80). The attributes and behaviours judgements were completed separately, with items presented in a random order.

#### Warmth and competence

Participants rated the target on their perceived warmth (warm, nice, friendly and sincere) and competence (competent, confident, skillful and able). Both measures were reliable across both the good listener face and bad listener face conditions (all *α*s > .83).

#### Humility

Humility was measured by adapting a scale developed by Owens *et al*. [80]. The items were reframed such that they referred to perceptions of another person’s humility (e.g. the item ‘I admit when I don’t know how to do something’ was rephrased to read ‘This person admits it when they don’t know how to do something’). Participants rated how well each item applied to the individual in the image, using a scale ranging from 1 (not at all) to 7 (very much). This measure showed high reliability across both conditions (*α*s > .93). This measure contained the attention check, an item where participants were required to respond 4.

#### Values

After completing the listener attributes and behaviours task, participants rated the image on their perceived values. Instead of having one item for each of Schwartz’s core values, we used four items per value type, using the examples presented in Study 1. We created a composite score for each value type (all *αs* > .71).

#### Self-perceived listening

Self-perceived listening was once again assessed via the AELS (*α* = .88).

#### Demographics

Participants finished the study by reporting their age, gender, education level, country of birth and country of residence.

### 7.2. Results

#### 7.2.1. Evaluations of good versus bad listener attributes and behaviours

Following our preregistration, we tested differences in evaluations of the good and bad listener faces. The results of these analyses are presented in table 4. Consistent with the results of Study 1, the good listener face was rated as significantly more likely to possess positive listening attributes and behaviours and significantly less likely to possess negative listening attributes and behaviours, relative to the bad listener face (all *ps* < .001, all *ds* > |0.75|).

**Table 4.** Evaluations of good and listening faces: Study 2.

	*good listener*	*bad listener*			
	*M* (s.d.)	*M* (s.d.)	*t*	Cohen’s *d*	*p*
positive attributes	5.22 (0.99)	3.43 (1.17)	16.12	1.64	<.001
negative attributes	2.84 (1.14)	3.77 (1.21)	−7.76	−0.79	<.001
positive behaviours	5.21 (0.98)	3.72 (1.18)	13.45	1.37	<.001
negative behaviours	2.51 (1.16)	3.75 (1.26)	−10.05	−1.03	<.001
warmth	5.44 (1.30)	3.11 (1.38)	16.96	1.73	<.001
competence	5.26 (0.97)	4.09 (1.07)	11.24	1.15	<.001
humility	4.99 (1.15)	3.26 (1.28)	13.97	1.42	<.001
self-transcendence	68.90 (18.36)	36.55 (22.85)	15.30	1.56	<.001
self-enhancement	57.87 (15.60)	45.57 (20.11)	6.71	0.68	<.001
openness	62.89 (15.31)	46.44 (18.24)	9.58	0.98	<.001
conservation	61.62 (16.06)	40.38 (21.55)	10.96	1.12	<.001

#### 7.2.2. Differences in perceptions of good versus bad listeners

The good listener face was judged as being warmer, more competent and more humble than the bad listener face (*p*s < .001, *ds* > 1.10). Regarding values, the good listener face was perceived as placing more importance on all four value types (all *ps* < .001, all *ds* > |0.65|). The effects are consistent with our hypotheses for self-transcendence and openness values, but opposite to our hypothesis for self-enhancement. The good listener face was also deemed to place greater importance on conservation values.

#### 7.2.3. Moderated mediation analyses

To examine whether condition impacted outcomes via positive and negative attributes and positive and negative behaviour scores, with a moderating role of AELS, we ran a series of moderated mediation analyses using Process Model 7 [63]. All relevant test statistics and confidence intervals are presented in table 5. As in Study 1, for parsimony, we focus on a verbal description of the results. We start by describing the effects of condition, AELS and their interaction on positive and negative attribute and behaviour scores, which are the same across all outcome variables, before discussing the effects on each outcome variable.

**Table 5.** Moderated mediation analyses for Study 2.

MEDIATOR VARIABLE REGRESSION MODELS									
	positive attributes			negative attributes					
	*b*	*t*	CI	*b*	*t*	CI			
condition	1.78^c^	16.19	[1.56, 1.99]	−0.92^c^	−7.73	[−1.16, −0.69]			
AELS	0.48^b^	2.25	[0.06, 0.90]	−0.26	−1.11	[−0.72, 0.20]			
cond * AELS	0.22	1.58	[−0.05, 0.49]	−0.14	−0.91	[−0.44, 0.16]			
	positive behaviours			negative behaviours					
	*b*	*t*	CI	*b*	*t*	CI			
condition	1.48^c^	13.51	[1.26, 1.69]	−1.24^c^	10.05	[−1.48, −1.00]			
AELS	0.54^b^	2.51	[0.12, 0.96]	−0.39	−1.63	[−0.87, 0.08]			
cond * AELS	0.25	1.84	[−0.02, 0.53]	−0.25	−1.6	[−0.56, 0.06]			
DEPENDENT VARIABLE REGRESSION MODELS									
	warmth			competence			humility		
	*b*	*t*	CI	*b*	*t*	CI	*b*	*t*	CI
direct effects									
positive attributes	0.78^c^	10.68	[0.64, 0.93]	0.48^c^	6.46	[0.33, 0.63]	0.50^c^	8.27	[0.38, 0.62]
negative attributes	−0.19^b^	−2.7	[−0.33, −0.05]	0.06	0.85	[−0.08, 0.20]	−0.19^b^	−3.23	[−0.30, −0.07]
positive behaviours	0.07	0.95	[−0.08, 0.23]	0.01	0.18	[−0.14, 0.17]	0.27^c^	4.15	[0.14, 0.40]
negative behaviours	0.07	0.92	[−0.07, 0.21]	−0.02	−0.34	[−0.17, 0.12]	−0.02	−0.28	[−0.13, 0.10]
condition	0.72^c^	6.08	[0.49, 0.96]	0.33^b^	2.67	[0.09, 0.56]	0.24^a^	2.43	[0.05, 0.43]
indirect effects									
Cond → Pos Attr → DV	1.39		[1.09, 1.71]	0.86		[0.56, 1.17]			
Cond → Neg Attr → DV	0.18		[0.01, 0.35]	−0.06		[−0.24, 0.11]			
Cond → Pos Beh → DV	0.11		[−0.16, 0.38]	0.02		[−0.22, 0.27]			
Cond → Neg Beh → DV	−0.09		[−0.33, 0.15]	0.03		[−0.19, 0.26]			
moderated mediation effects									
Cond → Pos Attr → DV	0.17		[−0.04, 0.40]	0.11		[−0.03, 0.25]			
Cond → Neg Attr → DV	0.03		[−0.03, 0.12]	−0.01		[−0.26, 0.12]			
Cond → Pos Beh → DV	0.02		[−0.03, 0.09]	0.00		[−0.04, 0.06]			
Cond → Neg Beh → DV	−0.02		[−0.09, 0.03]	0.01		[0.05, −0.07]			
	self-transcendence			self-enhancement					
	*b*	*t*	CI	*b*	*t*	CI			
direct effects									
positive attributes	10.11^c^	8.66	[7.82, 12.41]	6.69^c^	4.68	[3.88, 9.50]			
negative attributes	−2.61^a^	−2.34	[−4.80, −0.42]	3.13^a^	2.3	[0.45, 5,81]			
positive behaviours	1.97	1.57	[−0.49, 4.44]	0.09	0.06	[−2.93, 3.11]			
negative behaviours	−0.42	−0.36	[−2.66, 1.83]	1.61	1.16	[−1.13, 4.35]			
condition	8.42^c^	4.45	[4.79, 12.13]	5.15^a^	2.23	[0.60, 9.69]			
indirect effects									
Cond → Pos Attr → DV	17.99		[13.04, 23.51]	11.9		[6.94, 17.45]			
Cond → Neg Attr → DV	2.42		[0.04, 4.71]	−2.91		[−6.14, −0.08]			
Cond → Pos Beh → DV	2.91		[−1.07, 7.09]	0.13		[−4.56, 4.82]			
Cond → Neg Beh → DV	0.52		[−2.83, 3.82]	-2		[−6.01, 1.73]			
moderated mediation effects									
Cond → Pos Attr → DV	2.22		[−0.56, 5.37]	1.47		[−0.40, 3.59]			
Cond → Neg Attr → DV	0.36		[−0.51, 1.52]	0.44		[−1.78, 0.64]			
Cond → Pos Beh → DV	0.5		[−0.18, 1.77]	0.03		[−0.89, 1.07]			
Cond → Neg Beh → DV	0.11		[−0.73, 0.97]	−0.4		[−7.08, 2.08]			
	openness			conservation					
	*b*	*t*	CI	*b*	*t*	CI			
direct effects									
positive attributes	8.02^c^	6.37	[5.55, 10.49]	7.87^c^	6.59	[5.52, 10.21]			
negative attributes	4.75^c^	3.95	[2.38, 7.10]	−2.66^a^	−2.34	[−0.42, −4.90]			
positive behaviours	1.27	0.94	[−1.39, 3.93]	1.93	1.51	[−0.59, 4.46]			
negative behaviours	0.08	0.07	[−2.33, 2.50]	0.04	0.03	[−2.25, 2.32]			
condition	4.76^a^	2.34	[0.76, 8.76]	1.91	0.99	[−1.89, 5.70]			
indirect effects									
Cond → Pos Attr → DV	14.26		[9.18, 19.86]	13.99		[9.23, 19.25]			
Cond → Neg Attr → DV	−4.4		[−7.77, −1.73]	2.47		[0.16, 4.63]			
Cond → Pos Beh → DV	1.88		[−2.35, 6.09]	2.85		[−1.10, 6.93]			
Cond → Neg Beh → DV	−0.1		[−3.61, 3.16]	−0.04		[−3.08, 3.00]			
moderated mediation effects									
Cond → Pos Attr → DV	1.76		[−0.46, 4.33]	1.72		[−0.50, 4.02]			
Cond → Neg Attr → DV	−0.66		[−2.30, 0.91]	0.37		[−0.53, 1.46]			
Cond → Pos Beh → DV	0.33		[−0.37, 1.52]	0.49		[−0.22, 1.72]			
Cond → Neg Beh → DV	−0.02		[−0.99, 0.78]	0.01		[−0.85, 0.74]			

^a^ *p* < 0.05

^b^ *p* < 0.01

^c^ *p* < 0.001

#### *Effects of condition and AELS on attributes and listening behaviours (paths a*_*1*_ *to a*_*4*_*)*

First, for the path from condition to positive attributes (a_1_), there were significant effects of both conditions (*p* < .001) and AELS (*p* = .025). The interaction was non-significant (*p* = .115). For the path from condition to negative attributes (a_2_), there was a significant effect of condition (*p* < .001). The AELS and interaction effects were both non-significant (both *p*s > .260). For the path from condition to positive behaviours (a_3_), there were significant effects of both conditions (*p* < .001) and AELS (*p* = .013). The interaction was non-significant (*p* = .067). Finally, for the path from condition to negative behaviours (a_4_), there was a significant effect of condition (*p* < .001). The AELS and interaction effects were both non-significant (both *p*s > .100). Together, all paths showed direct effects of condition, all in the expected direction. There was a less consistent pattern regarding the role of AELS scores.

#### Warmth

The b_1_ path from positive listener attributes to warmth was significant (*p* < .001), as was the b_2_ path from negative listener attributes to warmth (*p* = .007). More positive listener attributes and less negative listener attributes were associated with greater warmth. The b_3_ and b_4_ behavioural paths were non-significant, both *p* > .340. The direct effect of condition on warmth was significant (*p* < .001).

Regarding indirect effects, the effect of condition on warmth via positive attributes, *b* = 1.39, S.E .= 0.16, 95% CI [1.09, 1.71], and negative attributes, *b* = 0.18, S.E. = 0.08, 95% CI [0.01, 0.35], were both significant. The effect of condition on warmth via positive behaviours and negative behaviours were both non-significant. There was no evidence of moderated mediation; the confidence intervals for all four indirect effects included zero.

#### Competence

The b_1_ path from positive listener attributes to competence was significant (*p* < .001), such that more positive listener attributes were associated with greater perceived competence. The b_2_, b_3_ and b_4_ paths were all non-significant, all *p* > .390. The direct effect from condition to competence was significant (*p* = .008).

Regarding indirect effects, the effect of condition on competence via positive attributes was significant, *b* = 0.86, S.E. = 0.15, 95% CI [0.56, 1.17]. The remaining indirect paths were all non-significant. There was no evidence of moderated mediation, the confidence intervals for all four indirect effects included zero.

#### Humility

The b_1_ path from positive listener attributes to humility was significant (*p* < .001), as was the b_2_ path from negative listener attributes (*p* = .001). More positive listener attributes and less negative listener attributes were associated with perceiving the target as more humble. The b_3_ path from positive listener behaviours to humility was significant (*p* < .001), such that positive listening behaviours were associated with perceiving the target as more humble. The b_4_ path from negative listener behaviours to humility was non-significant (*p* = .778). The direct effect of condition on humility was significant (*p* = .016).

Regarding indirect effects, the effect of condition on humility via positive attributes, *b* = 0.90, S.E .= 0.13, 95% CI [0.65, 1.18], negative attributes, *b* = 0.17, S.E. = 0.07, 95% CI [0.04, 0.33], and positive behaviours, *b* = 0.41, S.E. = 0.12, 95% CI [0.16, 0.64], were all significant. The effect of condition on humility via negative behaviours was non-significant. There was no evidence of moderated mediation, the confidence intervals for all four indirect effects included zero.

#### Self-transcendence

The b_1_ path from positive listener attributes to self-transcendence values was significant (*p* < .001), as was the b_2_ path from negative listener attributes (*p* = .020). More positive listener attributes and less negative listener attributes were associated with perceiving the target as placing greater importance on self-transcendence values. The b_3_ and b_4_ behavioural paths were non-significant, both *p* > .110. The direct effect of condition on self-transcendence values was significant (*p* < .001).

Regarding indirect effects, the effect of condition on self-transcendence values via positive attributes, *b* = 17.99, S.E. = 2.68, 95% CI [13.04, 23.51] and negative attributes, *b* = 2.42, S.E. = 1.18, 95% CI [0.04, 4.71], were both significant. The effects of condition on self-transcendence values via positive behaviours and negative behaviours were non-significant. There was no evidence of moderated mediation, the confidence intervals for all four indirect effects included zero.

#### Self-enhancement

The b_1_ path from positive listener attributes to self-enhancement values was significant (*p* < .001), as was the b_2_ path from negative listener attributes to self-enhancement values (*p* = .022). More positive *and* more negative listener attributes were associated with perceiving the target as placing greater importance on self-enhancement values. The b_3_ and b_4_ behavioural paths were non-significant. The direct effect of condition on self-enhancement values was significant (*p* = .027).

Regarding indirect effects, the effect of condition on self-enhancement values via positive attributes, *b* = 11.90, S.E .= 2.71, 95% CI [6.94, 17.45], and negative attributes, *b* = −2.91, S.E. = 1.55, 95% CI [−6.14, −0.08], were both significant. The effect of condition on self-enhancement values via positive behaviours and negative behaviours were both non-significant. There was no evidence of moderated mediation, the confidence intervals for all four indirect effects included zero.

#### Openness

The b_1_ path from positive listener attributes to openness values was significant (*p* < .001), as was the b_2_ path from negative listener attributes (*p* < .001). More positive *and* more negative listener attributes were associated with perceiving the target as placing greater importance on openness values. The b_3_ and b_4_ behavioural paths were non-significant, both *p* > .340. The direct effect of condition on self-transcendence values was significant (*p* = .020).

Regarding indirect effects, the effect of condition on openness values via positive attributes, *b* = 14.26, S.E .= 2.69, 95% CI [9.18, 19.86], and negative attributes, *b* = −4.40, S.E. = 1.50, 95% CI [−7.77, −1.73], were both significant. The effect of condition on openness values via positive behaviours and negative behaviours were both non-significant. There was no evidence of moderated mediation, the confidence intervals for all four indirect effects included zero.

#### Conservation

For these exploratory analyses, the b_1_ path from positive listener attributes to openness values was significant (*p* < .001), as was the b_2_ path from negative listener attributes (*p* < .001). More positive *and* less negative listener attributes were associated with perceiving the target as placing greater importance on conservation values. The b_3_ and b_4_ behavioural paths were non-significant, both *p* > .130. The direct effect of condition on self-transcendence values was non-significant (*p* = .324).

Regarding indirect effects, the effect of condition on conservation values via positive attributes, *b* = 13.99, S.E .= 2.56, 95% CI [9.23, 19.25], and negative attributes, *b* = 2.47, S.E. = 1.13, 95% CI [0.16, 4.63], were both significant. The effect of condition on openness values via positive behaviours and negative behaviours were both non-significant. There was no evidence of moderated mediation, the confidence intervals for all four indirect effects included zero.

#### 7.2.4. Correcting for multiple comparisons

As in Study 1, we employed the Benjamini–Hochberg procedure [64] to correct for multiple comparisons. In total, 41 statistical tests were included in the analysis, namely, *t*-tests, main effects, indirect effects and moderated mediation effects. The original *p*-values ranged from .001 ≤ *p* ≤ .980, and for significant tests, the range was .001 ≤ *p* ≤ .022. After applying the B–H correction, adjusted *p*-values were calculated to control the false discovery rate at a 5% threshold. All significant tests remained significant after correction, with adjusted *p*-values ranging from .001 ≤ *p* ≤ .047. That is, no original significant test became non-significant after the B–H correction.

#### 7.2.5. Discussion

Study 2 replicated the results of Study 1 and provided initial support for hypothesis 4, namely, participants judged the good listener’s face as more humble than the bad listener’s face. However, contrary to hypothesis 6, the good listener image was associated with higher self-enhancement values than the bad listener, which was unexpected. While the experimental condition influenced the mediators such as positive and negative attributes, no moderation by AELS was observed in Study 2. This contrasts with Study 1, where stronger mediation effects were found among participants who perceived themselves as good listeners. The difference might be because, in Study 1, participants were evaluating a known acquaintance, which could have intensified the influence of their self-perceived listening abilities on their judgements of others. Overall, Study 2 offers new insights into how people visualize and evaluate good versus bad listeners, highlighting the strong association between these mental representations and their evaluations.

Despite the general support for our model in Studies 1 and 2, an alternative explanation for the effects on the outcome variables is that a good listener creates a Halo effect, increasing positive features and decreasing negative ones. However, a Halo effect does not explain why we observed a positive effect of the good listener condition in Study 2 on self-enhancement values, which are typically rated as the least important (i.e. desirable) value type [31]. Moreover, in Studies 1 and 2 the downstream effect was mediated by listening attributes and behaviours. Yet, we believe a more robust test is needed to refute the possibility of a Halo effect. Therefore, we conducted Study 3.

## 8. Study 3

Study 3 was designed to conceptually replicate and extend Study 2 by testing whether the good and bad listener classification images would elicit unique effects compared with another set of classification images. Such a pattern would imply that there is something special about the good and bad listener classification images linking them to listening attributes and behaviours. Put differently, we sought to distil the effects of the listener faces from any valence attributable to positive and negative classification images derived from using a construct linked with listening. Participants were randomly assigned to evaluate one of four classification images—the good or bad listener faces from Study 2, or classification images of a non-narcissist or narcissist that were generated in a separate project by Smith *et al*. [74], where 100 participants provided their representation of a narcissist, with narcissist and non-narcissist classification images derived following a procedure described by Brown-Iannuzzi *et al*. [81]. We selected narcissism because (i) non-narcissists and narcissists are evaluated differently on a range of outcomes, including warmth and competence (74), and (ii) evidence linking narcissism with bad listening [82,83]. As such, the classification images differ on two dimensions—face type (listening versus narcissism) and face valence (positive (good listener/non-narcissist) versus negative (bad listener/narcissist)).

As in Study 2, the hypotheses, dependent variables, analyses, sample size and exclusion criteria were preregistered at: https://aspredicted.org/5SY_VCY. We did not preregister the moderated mediation analysis given the extreme number of moderated mediation indexes (over 160) in the design (2 × 2 between participants, with four mediators and six outcomes), which would make any inferences problematic.

### 8.1. Method

#### 8.1.1. Participants

A total of 542 participants (*M*_age_ = 32.48 years; s.d. = 10.55) were recruited via Prolific. Participants were paid £1.06 for their participation. No participants failed the attention check. Sensitivity analysis indicated that the smallest effect size that this sample size can detect with a power of 80% (two-tailed) and *α* = .05 is Cohen’s *f* = 0.14 [56].

#### 8.1.2. Procedure

**Figure 5.**
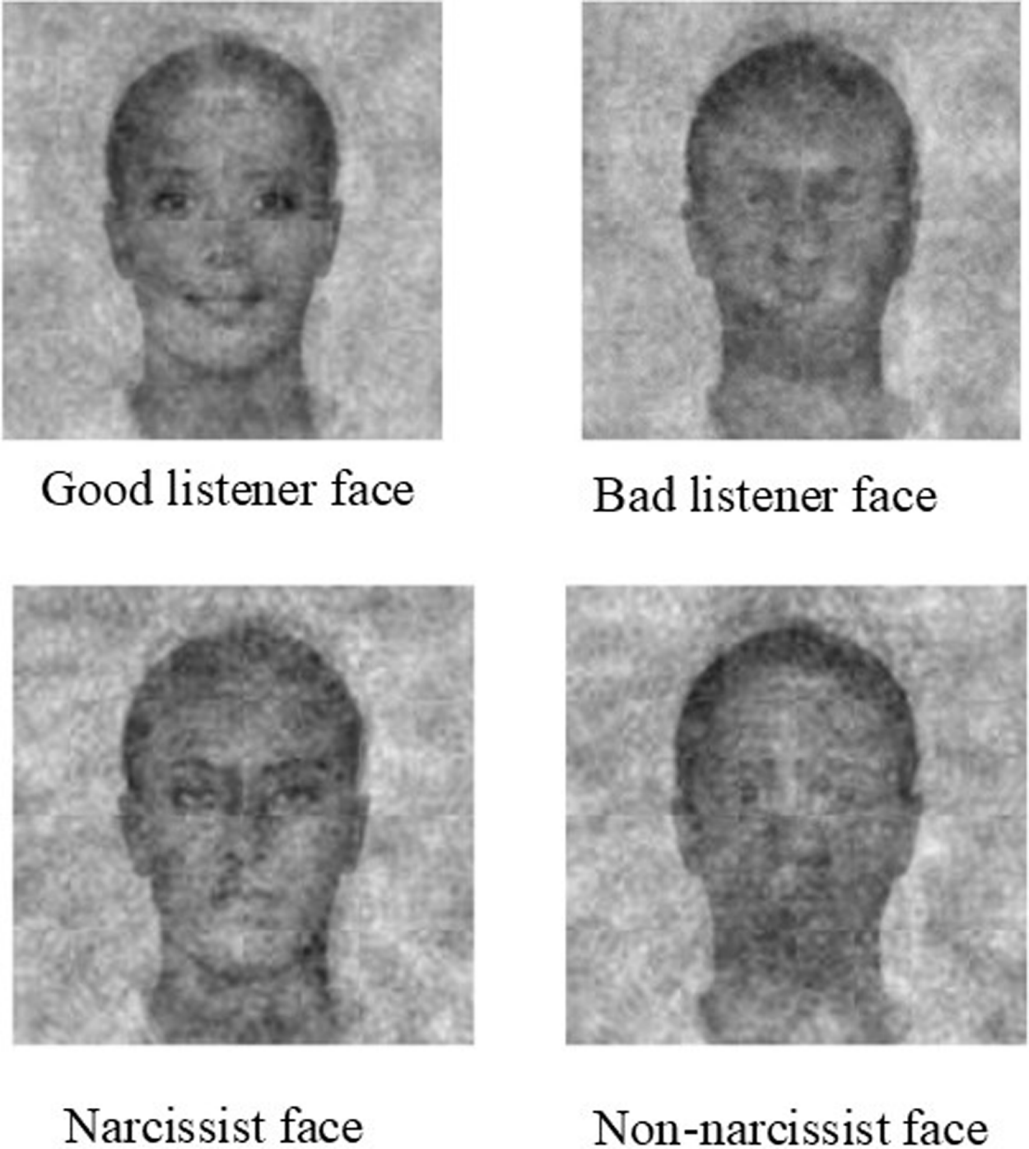
Good listener, bad listener, narcissist and non-narcissist faces.

Like in Study 2, we informed participants that they would be making judgements about a visually distorted image. Participants were randomly assigned to evaluate either the good or bad listener image, or either the narcissist or non-narcissist image. The narcissist and non-narcissist images were created using the same base face as the good and bad listener faces. All four faces are presented in figure 5. Participants evaluated their assigned face using the same measures as in Study 2 and also completed the AELS. Like Study 2, an attention check item was included in the humility measure.

### 8.2. Measures

**Listener attributes and behaviours.** As in Study 2, we used participants’ responses to compute four indices: positive listening attributes, negative listening attributes, positive listening behaviours and negative listening behaviours. Each index showed high reliability across all conditions (.74 ≤ *αs* ≤ .93). The attributes and behaviours judgements were completed separately, with items presented in a random order.

**Warmth and competence.** We measured warmth (.70 ≤ *αs* ≤ .90) and competence (.93 ≤ *αs* ≤ .96) with the same scales as in Study 2.

**Values.** We measured values with the same scales as Study 2 (.70 ≤ *αs* ≤ .90).

**Humility.** We used the same measure as in Study 2 (.94 ≤ *αs* ≤ .96).

**Self-perceived listening.** As in Studies 1 and 2, self-perceived listening was assessed via the AELS (*α* = .87).

**Demographics.** Participants finished the study by reporting their age, gender, education level, country of birth and country of residence.

### 8.3. Results

We tested our hypotheses via a set of 2 (face type: listener versus narcissist) by 2 (face valence: positive versus negative) ANOVAs. These analyses are summarized in table 6. For parsimony, we focus on the face type by face valence interaction. The main effects and interaction effects are presented in table 6 as well as effect sizes for the interactions.

**Table 6.** ANOVA summary of Study 3.

	good listener	bad listener	non-narcissist	narcissist	main effect: face type	main effect: face valence	interaction effect	
	**Mean**	**Mean**	**Mean**	**Mean**	***F* (1,529**)	***F* (1,529**)	***F* (1,529**)	***Cohen’s f***
positive attributes	5.40	3.60	4.00	3.56	50.45, *p* < .001	122.18, *p* < .001	44.85, *p* < .001	0.29
negative attributes	2.93	3.68	3.67	3.77	15.67, *p* < .001	16.63, *p* < .001	9.50, *p* < .001	0.13
positive behaviours	5.30	3.92	4.19	3.76	45.54, *p* < .001	89.89, *p* < .001	25.16, *p* < .001	0.22
negative behaviours	2.62	4.50	3.47	3.33	9.52, *p* = .002	11.31, *p* = .001	21.49, *p* < .001	0.20
warmth	5.64	3.48	4.14	2.60	89.01, *p* < .001	214.66, *p* < .001	6.02, *p* = .015	0.11
competence	5.50	4.23	4.51	4.75	4.67, *p* = .031	23.45, *p* < .001	49.08, *p* < .001	0.30
humility	5.25	3.48	4.08	3.06	45.77, *p* < .001	142.78, *p* < .001	10.49, *p* = .001	0.14
self-transcendence	71.07	41.47	49.81	38.88	38.73, *p* < .001	111.71, *p* < .001	23.72, *p* < .001	0.21
self- enhancement	62.56	45.33	48.69	56.33	0.65, *p* = .421	7.16, *p* = .008	48.28, *p* < .001	0.30
openness	64.91	46.87	52.10	43.88	21.60, *p* < .001	59.68, *p* < .001	8.35, *p* = .004	0.12
conservation	66.47	43.49	51.38	39.09	29.08, *p* < .001	95.28, *p* < .001	8.76, *p* = .003	0.13

On the measures of positive and negative listening attributes and behaviours, all of the 2 × 2 ANOVAs revealed significant face type by face valence interactions (all *p* < .01, all Cohen’s *f* > 0.12). In all cases, the difference in evaluations between the listener faces was significantly greater than the difference in evaluations between the narcissism faces, with all effect sizes at least three times larger for the listening faces relative to the narcissism faces.

On the measures of warmth, competence, and humility, all of the 2 × 2 ANOVAs revealed significant face type by face valence interactions (all *p* < .05, all Cohen’s *f* > 0.09). As with the attributes and listening behaviours, the difference in evaluations between the listener faces was significantly greater than the difference in evaluations between the narcissism faces.

Regarding self-transcendence and openness values, the effects were in the expected direction and larger for the listener faces compared with the narcissism faces. The good listener face was seen as espousing self-enhancement values more strongly compared with the bad listener face. Further, this effect was larger than that observed for the narcissism faces. A similar pattern was found for conservation values.

#### 8.3.1. Moderated mediation analyses

We conducted moderated mediation analyses using the same approach as in Studies 1 and 2 (PROCESS Model 7 [63]). Because our independent variable for these analyses was categorical, and we did not assume linearity between the experimental groups, we used an indicator coding scheme [84]. An indicator coding scheme also allows for a separate comparison of the indirect effects of the good listener face and each of the other groups while controlling for the other main effects.

As in Studies 1 and 2, we summarize the results for each outcome variable. Of course, the mediation analyses become more complicated given the need to use an indicator coding scheme, which increased the number of tested effects. Regarding moderated mediation, because all indices of moderated mediation were non-significant, these effects are not discussed any further.

#### Warmth

When comparing the good listener face with the non-narcissist face the indirect effect through positive attributes was significant, *b* = 1.45, S.E. = 0.15, 95% CI [1.17, 1.76]. The indirect effect through negative attributes was also significant, *b* = 0.13, S.E. = 0.06, 95% CI [0.01, 0.26]. The indirect effect through positive behaviours was significant, *b* = 0.29, S.E. = 0.11, 95% CI [0.08, 0.51], as was the indirect effect through bad behaviours, *b* = −0.14, S.E. = 0.05, 95% CI [−0.26, −0.05]. The direct effect was significant, *b* = 1.27, S.E. = 0.14*, t* = 8.92, *p* < .001, 95% CI [0.99, 1.55].

In the comparison between the good listener and the narcissist faces, the indirect effect through positive attributes was significant, *b* = 1.11, S.E. = 0.13, 95% CI [0.86, 1.39]. The indirect effect through negative attributes was significant, *b* = 0.11, S.E. = 0.06, 95% CI [0.01, 0.23]. The indirect effect through positive behaviours was significant, *b* = 0.21, S.E = 0.08, 95% CI [0.06, 0.37]. The indirect effect through negative behaviours was significant, *b* = −.0..17, S.E. = 0.06, 95% CI [−0.30, −0.06]. The direct effect was not significant, *b* = 0.21, S.E .= 0.13, *t* = 1.63, *p* = .104, 95% CI [−0.04, 0.47].

Finally, when comparing the good listener face with the bad listener face, the indirect effect through positive attributes was significant, *b* = 1.41, S.E .= 0.16, 95% CI [1.11, 1.73]. The indirect effect through negative attributes was significant, *b* = 0.11, S.E. = 0.06, 95% CI [0.01, 0.22]. The indirect effect through positive behaviours was significant, *b* = 0.26, S.E. = 0.10, 95% CI [0.07, 0.44]. The indirect effect through negative behaviours was significant, *b* = −0.17, S.E. = 0.06, 95% CI [−0.30, −0.06]. The direct effect was significant, *b* = 0.50, S.E. = 0.14, *t* = 3.62, *p* < .001, 95% CI [0.23, 0.77].

#### Competence

When comparing the good listener face with the non-narcissist face the indirect effect through positive attributes was significant, *b* = 0.86, S.E. = 0.13, 95% CI [0.61, 1.13]. The indirect effect through negative attributes was also significant, *b* = −0.14, S.E. = 0.06, 95% CI [−0.26, −0.04]. The indirect effect through positive behaviours was significant, *b* = 0.38, S.E. = 0.11, 95% CI [0.18, 0.61]. The indirect effect through negative behaviours was not significant, *b* = 0.0.2, S.E = 0.05, 95% CI [−0.06, 0.12]. The direct effect was also significant, *b* = −0.40, S.E. = 0.15, *t* = −2.75, *p* = .006, 95% CI [−0.69, −0.11].

When comparing the good listener face with the narcissist face, the indirect effect through positive attributes was significant, *b* = 0.66, S.E. = 0.11, 95% CI [0.46, 0.88]. The indirect effect through negative attributes was significant, *b* = −0.12, S.E. = 0.05, 95% CI [−0.22, −0.03]. The indirect effect through positive behaviours was significant, *b* = 0.28, S.E. = 0.08, 95% CI [0.13, 0.44]. The indirect effect through negative behaviours was not significant, *b* = 0.03, S.E. = 0.05, 95% CI [−0.08, 0.14]. The direct effect was not significant, *b* = 0.13, S.E. = 0.13, *t* = 0.96, *p* = .340, 95% CI [−0.14, 0.39].

Finally, when comparing the good listener face with the bad listener face, the indirect effect through positive attributes was significant, *b* = 0.84, S.E. = 0.12, 95% CI [0.60, 1.08]. The indirect effect through negative attributes was significant, *b* = −0.12, S.E. = 0.05, 95% CI [−0.23, −0.03]. The indirect effect through positive behaviours was significant, *b* = 0.34, S.E. = 0.10, 95% CI [0.16, 0.54]. The indirect effect through negative behaviours was not significant, *b* = 0.03, S.E. = 0.05, 95% CI [−0.07, 0.14]. The direct effect was not significant, *b* = 0.16, S.E = 0.14, *t* = 1.12, *p* = .263, 95% CI [−0.12, 0.44].

#### Humility

When comparing the good listener face with the non-narcissist face, the indirect effect through positive attributes was significant, *b* = 1.10, S.E. = 0.14, 95% CI [0.84, 1.37]. Unlike the other constructs, the indirect effect through negative attributes was not significant, *b* = 0.08, S.E. = 0.05, 95% CI [−0.01, 0.19]. The indirect effect through positive behaviours was significant, *b* = 0.49, S.E. = 0.11, 95% CI [0.29, 0.71]. The indirect effect through negative behaviours was not significant, *b* = −0.03, S.E. = 0.04, 95% CI [−0.12, 0.05]. The direct effect was not significant, *b* = 0.50, .S.E. = 0.12, *t* = 4.13, *p* < .001, 95% CI [0.26, 0.74].

When comparing the good listener face with the narcissist face, the indirect effect through positive attributes was significant, *b* = 0.85, S.E.= 0.11, 95% CI [0.63, 1.07]. The indirect effect through negative attributes was not significant, *b* = 0.07, S.E. = 0.05, 95% CI [−0.01, 0.17]. The indirect effect through positive behaviours was significant, *b* = 0.36, S.E. = 0.08, 95% CI [0.21, 0.53]. The indirect effect through negative behaviours was not significant, *b* = −0.04, S.E. = 0.05, 95% CI [−0.13, 0.06]. The direct effect was not significant, *b* = −0.09, S.E. = 0.11, *t* = 0.84, *p* = .399, 95% CI [−0.31, 0.12].

Finally, when comparing the good listener face with the bad listener face, the indirect effect through positive attributes was significant, *b* = 1.07, *S.E.* = 0.13, 95% CI [0.82, 1.34]. The indirect effect through negative attributes was not significant, *b* = 0.07, S.E. = 0.04, 95% CI [−0.01, 0.16]. The indirect effect through positive behaviours was significant, *b* = 0.43, S.E .= 0.10, 95% CI [0.26, 0.64]. The indirect effect through negative behaviours was not significant, *b* = −0.03, S.E .= 0.05, 95% CI [−0.13, 0.06]. The direct effect was not significant, *b* = 0.18, S.E. = 0.12, *t* = 1.55, *p* = .123, 95% CI [−0.05, 0.41].

#### Self-transcendence

When comparing the good listener face with the non-narcissist face, the indirect effect through positive attributes was significant, *b* = 18.96, S.E .= 2.32, 95% CI [14.52, 23.76]. The indirect effect through negative attributes was also significant, *b* = 2.67, S.E .= 0.99, 95% CI [0.96, 4.84]. The indirect effect through positive behaviours was significant, *b* = 3.77, S.E. = 1.78, 95% CI [0.42, 7.35]. The indirect effect through negative behaviours was not significant, *b* = −1.04, S.E. = 0.73, 95% CI [−2.65, 0.24]. The direct effect was also significant, *b* = 7.19, S.E. = 2.23, *t* = 3.23, *p* = .001, 95% CI [2.81, 11.58].

When comparing the good listener face with the narcissist face, the indirect effect through positive attributes was significant, *b* = 14.53, S.E. = 1.92, 95% CI [10.89, 18.32]. The indirect effect through negative attributes was significant, *b* = 2.33, S.E .= 0.85, 95% CI [0.85, 4.19]. The indirect effect through positive behaviours was significant, *b* = 2.74, S.E. = 1.28, 95% CI [0.32, 5.31]. The indirect effect through negative behaviours was not significant, *b* = −1.28, S.E. = 0.85, 95% CI [−3.12, 0.29]. The direct effect was not significant, *b* = 2.57, S.E. = 2.05, *t* = 1.26, *p* = .209, 95% CI [−1.45, 6.60].

Finally, when comparing the good listener face with the bad listener face, the indirect effect through positive attributes was significant, *b* = 18.44, S.E. = 2.37, 95% CI [13.99, 23.20]. The indirect effect through negative attributes was significant, *b* = 2.25, S.E. = 0.85, 95% CI [0.81, 4.11]. The indirect effect through positive behaviours was significant, *b* = 3.34, S.E. = 1.57, 95% CI [0.37, 6.44]. The indirect effect through negative behaviours was not significant, *b* = −1.27, S.E. = 0.85, 95% CI [−3.03, 0.28]. The direct effect was significant, *b* = 6.02, S.E. = 2.17, *t* = 2.77, *p* = .006, 95% CI [1.75, 10.29].

#### Self-enhancement

When comparing the good listener face with the non-narcissist face the indirect effect through positive attributes was significant, *b* = 10.44, S.E. = 2.50, 95% CI [5.74, 15.41]. The indirect effect through negative attributes was also significant, *b* = −3.85, S.E. = 1.19, 95% CI [−6.32, −1.71]. The indirect effect through positive behaviours was significant, *b* = 5.51, S.E. = 2.24, 95% CI [1.41, 10.22]. The indirect effect through negative behaviours was not significant, *b* = 0.01, S.E. = 0.81, 95% CI [−1.72, 1.56]. The direct effect was significant, *b* = −6.21, S.E. = 2.73*, t* = −2.28, *p* = .023, 95% CI [−11.57, −0.85].

When comparing the good listener face with the narcissist face, the indirect effect through positive attributes was significant, *b* = 8.01, S.E. = 2.02, 95% CI [4.24, 12.19]. The indirect effect through negative attributes was significant, *b* = −3.36, S.E. = 1.10, 95% CI [−5.68, −1.43]. The indirect effect through positive behaviours was significant, *b* = 4.00, S.E. = 1.65, 95% CI [0.99, 7.54]. The indirect effect through negative behaviours was not significant, *b* = 0.02, S.E. = 0.98, 95% CI [−2.04, 1.84]. The direct effect was significant, *b* = 5.07, S.E. = 2.50, *t* = 2.03, *p* = .043, 95% CI [0.15, 9.99].

Finally, when comparing the good listener face with the bad listener face, the indirect effect through positive attributes was significant, *b* = 10.16, S.E. = 2.46, 95% CI [5.55, 15.14]. The indirect effect through negative attributes was significant, *b* = −3.24, S.E. = 1.06, 95% CI [−5.53, −1.36]. The indirect effect through positive behaviours was significant, *b* = 4.87, S.E. = 2.01, 95% CI [1.21, 9.13]. The indirect effect through negative behaviours was not significant, *b* = 0.02, S.E. = 0.97, 95% CI [−2.07, 1.84]. The direct effect was not significant, *b* = 5.19, S.E. = 2.66, *t* = 1.95, *p* = .051, 95% CI [−0.03, 10.41].

#### Openness

When comparing the positive listener face with the non-narcissist face the indirect effect through positive attributes was significant, *b* = 13.62, S.E. = 2.30, 95% CI [9.22, 18.21]. The indirect effect through negative attributes was significant, *b* = −2.74, S.E. = 1.01, 95% CI [−4.91, −0.90]. The indirect effect through positive behaviours was significant, *b* = 5.94, S.E. = 2.08, 95% CI [2.00, 10.06]. The indirect effect through negative behaviours was not significant, *b* = −1.34, S.E. = 0.83, 95% CI [−3.17, 0.08]. The direct effect was significant, *b* = 5.18, S.E. = 2.45*, t* = 2.12, *p* = .035, 95% CI [0.37, 10.00].

In the pairwise comparison between the good listener and the narcissist faces, the indirect effect through positive attributes was significant, *b* = 10.44, S.E .= 1.90, 95% CI [6.91, 14.43]. The indirect effect through negative attributes was significant, *b* = −2.39, S.E. = 0.93, 95% CI [−4.41, −0.74]. The indirect effect through positive behaviours was significant, *b* = 4.32, S.E. = 1.51, 95% CI [1.51, 7.37]. The indirect effect through negative behaviours was not significant, *b* = −1.65, S.E. = 0.96, 95% CI [−3.66, 0.10]. The direct effect was not significant, *b* = 1.94, S.E. = 2.25, *t* = 0.86, *p* = .389, 95% CI [−2.48, 6.36].

Finally, when comparing the good listener face with the bad listener face, the indirect effect through positive attributes was significant, *b* = 13.25, S.E. = 2.30, 95% CI [8.95, 18.04]. The indirect effect through negative attributes was significant, *b* = −2.31, S.E. = 0.93, 95% CI [−4.36, −0.70]. The indirect effect through positive behaviours was significant, *b* = 5.26, S.E. = 1.88, 95% CI [1.76, 9.02]. The indirect effect through negative behaviours was not significant, *b* = −1.63, S.E. = 0.97, 95% CI [−3.69, 0.11]. The direct effect was not significant, *b* = 3.20, S.E. = 2.39, *t* = 1.34, *p* = .180, 95% CI [−1.49, 7.89].

#### Conservation

For these exploratory analyses, when comparing the positive listener face with the non-narcissist face the indirect effect through positive attributes was significant, *b* = 16.80, S.E. = 2.42, 95% CI [12.23, 21.61]. The indirect effect through negative attributes was significant, *b* = 1.92, S.E. = 0.96, 95% CI [0.27, 4.02]. The indirect effect through positive behaviours was not significant, *b* = 2.36, S.E. = 1.79, 95% CI [−1.01, 6.12]. The indirect effect through negative behaviours was not significant, *b* = −0.23, S.E. = 0.69, 95% CI [−1.65, 1.09]. The direct effect was significant, *b* = 5.95, S.E. = 2.25*, t* = 2.65, *p* = .008, 95% CI [1.53, 10.36].

In the pairwise comparison between the good listener and the narcissist faces, the indirect effect through positive attributes was significant, *b* = 12.88, S.E. = 2.01, 95% CI [9.01, 16.99]. The indirect effect through negative attributes was significant, *b* = 1.68, S.E. = 0.83, 95% CI [0.24, 3.48]. The indirect effect through positive behaviours was not significant, *b* = 1.72, S.E. = 1.31, 95% CI [−0.73, 4.50]. The indirect effect through negative behaviours was not significant, *b* = −0.29, S.E. = 0.84, 95% CI [−2.00, 1.34]. The direct effect was not significant, *b* = −1.23, S.E. = 2.06, *t* = −0.60, *p* = .550, 95% CI [−5.29, 2.82].

Finally, when comparing the good listener face with the bad listener face, the indirect effect through positive attributes was significant, *b* = 16.34, S.E. = 2.43, 95% CI [11.69, 21.21]. The indirect effect through negative attributes was significant, *b* = 1.62, S.E. = 0.82, 95% CI [0.23, 3.44]. The indirect effect through positive behaviours was not significant, *b* = 2.09, S.E. = 1.59, 95% CI [−0.90, 5.38]. The indirect effect through negative behaviours was not significant, *b* = 1.63, S.E. = 0.97, 95% CI [−3.69, 0.11]. The direct effect was not significant, *b* = 2.47, S.E. = 2.19, *t* = 1.13, *p* = .259, 95% CI [−1.83, 6.77].

We found a highly similar pattern of mediation when comparing the bad listener face with all other faces. Positive and negative attributes, along with positive behaviours, mediated the effects on our outcome variables, with the only exception being the role of negative attributes on humility.

### 8.4. Correcting for multiple comparisons

As in Studies 1 and 2, the Benjamini–Hochberg procedure was employed to correct for multiple comparisons. In total, 63 statistical tests were included in the B–H analysis for Study 3. These tests comprised *t*-tests, ANOVAs, main effects, interactions, mediation effects and moderated mediation effects. The original *p*-values ranged from .001 ≤ *p* ≤ .985, with significant *p*-values ranging from .001 ≤ *p* ≤ .034. After applying the B–H correction, all originally significant tests (*p* < .05) remained significant, with adjusted *p*-values ranging from .001 ≤ *p* ≤ .048. As in the previous studies, no tests became non-significant after the correction.

### 8.5. Discussion

Study 3 replicated the effects observed in Study 2, with the good listener face being perceived more positively than the bad listener face. The study also ruled out the possibility that these effects were due to a general positive valence (Halo effect), as the effects were distinct from those related to narcissism. The good listener face was consistently associated with more positive listening attributes and behaviours, greater warmth, competence, humility and stronger self-transcendence and openness values. Additionally, the effects were mediated through positive attributes, negative attributes (except for humility) and positive behaviours, but not negative behaviours (except for warmth).

## 9. General discussion

Listening is a crucial element in social relationships, fundamental to building connections, fostering intimacy and resolving conflicts. Despite its importance in everyday interactions, there remains a significant gap in our understanding of how listeners are perceived and evaluated by others. Our research addresses this gap by exploring how people form perceptions and judgements about listeners.

We found consistent support for our hypotheses that good listeners are perceived as warmer, more competent and more humble relative to bad listeners. Good listeners were also judged as attaching greater importance to self-transcendence and openness values compared with bad listeners, with less consistent effects on self-enhancement values. As far as we are aware, these studies represent the first empirical evidence linking listening and values.

We also tested whether the attributes and behaviours of good and bad listeners explained the effect of listening perception on the outcome variables, finding supporting evidence of mediation. In Study 1, we found that the attributes and behaviours participants associated with a good or bad listener acquaintance mediated our outcome variables. In Study 2, where participants evaluated a classification image of a good or bad listener, we separated the valence of listening attributes and behaviours and found consistent evidence regarding the mediating role of positive and negative attributes on our outcome variables. In Study 3, in our analyses where we compared the good listener face with all faces, we found consistent mediating effects of positive attributes, negative attributes and positive behaviours, with no mediation through negative behaviours (aside from warmth). Taken together, the results suggest that listening attributes are a particularly meaningful mediator across a range of downstream effects.

Studies 2 and 3 address the central question of how people perceive listeners by using the reverse correlation method to reveal participants’ implicit mental representations of good and bad listeners. This method provides a visual representation of how people conceptualize listening qualities by allowing participants to select facial images that they associate with a *good* or *bad* listener. These classification images were then evaluated by a second sample on listening-specific attributes and behaviours (e.g. attentiveness, empathy, interrupting), uncovering the traits people implicitly link with listening competence. The reverse correlation approach is particularly valuable for studying listening because it captures implicit perceptions that participants might struggle to articulate explicitly. By examining how people associate visual cues with listening-related attributes and behaviours, the studies illuminate the underlying cognitive processes involved in listener perception. This method demonstrates that people perceive good listeners as embodying positive listening attributes (e.g. warmth, attentiveness) and behaviours (e.g. paying attention, showing empathy) while associating bad listeners with negative attributes and behaviours. In doing so, the reverse correlation technique provides a novel and listening-specific insight into how individuals form impressions of listeners.

Our tests of moderated mediation showed effects that differed as a function of whether the target was someone selected by the participant (as in Study 1) or an unknown classification image that was presented without any diagnostic information (as in Studies 2 and 3). While the former approach showed evidence of moderated mediation, with stronger mediation among participants who perceived themselves as good listeners, the latter approach showed no evidence of moderated mediation. Though speculative, one explanation for this difference is that the relationship between participants and their known acquaintances may have served to magnify how participants judge others whom they see as possessing (or not) attributes that they believe they possess [51,52].

Aside from this multi-method approach, another important methodological contribution of the research reflects its use of bottom-up processes, where Study 1 participants generated their own responses of what constitutes good or bad listeners, rather than using a top-down process where participants evaluated dimensions taken from existing listening scales [53,85]. The responses generated by participants were then used in Studies 2 and 3, where raters blindly evaluated classification images that themselves were generated by another sample. As such, the studies offer novel insights into how lay participants conceptualize good versus bad listeners.

An important theoretical contribution derived from our research is that the findings shed light on the *prestige* associated with being a good listener. Indeed, the mere perception that someone is a good listener strongly influences judgements about personality attributes and values that are considered desired and are core to social perception (see [30,31]). Even when a perceiver has never interacted with a target and has no diagnostic information about the target, we found strong effects on a range of outcome variables. From our perspective, the findings obtained via the reverse correlation paradigm are particularly informative and build upon research demonstrating that when simply seeing a face, participants make accurate judgements about targets’ emotions and attributes (e.g. [86,87]). The strong main effects of these studies might help explain why people may become more eager or reluctant to engage in conversation with a stranger they immediately perceive as a good or bad listener.

Our research builds on Bodie *et al*. [15] by exploring the fundamental question of how listeners are perceived, while also making several theoretical contributions. First, our research included assessments of both poor and good listeners, unlike Bodie *et al*. [15], which focused solely on good listeners. Second, while Bodie *et al*. [15] examined listening within the context of initial interactions, our study adopted a broader perspective, encompassing listeners across various contexts. Third, we extended the investigation to examine the downstream consequences of these perceptions, specifically how warmth, competence and values are associated with listener evaluations. Fourth, we explored how individuals' self-perceptions of their listening quality influenced the valence they assigned to the attributes and behaviours of good and bad listeners. These latter contributions formed the basis of our moderated mediation model, which provides a novel theoretical framework for understanding how people perceive listeners.

The findings from Studies 2 and 3, which relied on evaluating images, suggest that individuals may have prototypical visual images of good and bad listeners, which are shaped, in part, by facial expressions and other visual cues. These visual cues may influence how listeners are perceived. While these studies contribute to our understanding of the role visual cues play in forming these mental images, it is important to clarify that the primary contribution of Studies 2 and 3 may lie more in highlighting the implicit nature of these visual evaluations, rather than offering a comprehensive understanding of how people explicitly evaluate listeners. These studies reveal that visual cues play a crucial role in shaping our perceptions of listening quality, but the scope of this contribution may be more about uncovering the prototypical images people have, rather than providing in-depth insights into the cognizant psychological process of evaluating good or bad listeners.

### 9.1. Limitations and future research

The current research has several limitations that future research could address. Although our samples consisted of non-students, which offers a more representative view of society compared with undergraduate student samples (who are often criticized for their limited generalizability, see [88,89]), they were predominantly from WEIRD nations [89,90]. Future research should explore how listeners are perceived across an even more diverse range of participants. Given different cross-cultural norms regarding listening style preferences and interpersonal communication [91,92], it is conceivable that different attributes and behaviours might be associated with good and/or bad listeners across cultures.

Second, because the goal of the present study was to test how listeners are perceived, it overlooks the nuances of real-life listening experiences. For example, an individual might be initially perceived as a good listener yet fail to address the underlying emotional nuances of the speaker.

Third, the discrepancy in moderated mediation effects, where Study 1 showed significant effects with known acquaintances, while Studies 2 and 3 did not with unknown images, is an empirical limitation. This variation might suggest that personal relationships influence how listening attributes are perceived and judged, potentially amplifying effects when participants are familiar with the targets. Future research should explore how different relationship types impact listening perceptions by including a broader range of familiarity levels and controlling for relationship variables such as level of intimacy and satisfaction.

Fourth, in studies 2 and 3 participants assessed images of faces on the extent to which they resembled good or bad listeners, using a set of attributes and behaviours. To examine our theoretical model, we combined these items into four indices—positive and negative attributes and behaviours. Therefore, we did not examine individual attributes and behaviours. Future research can address this question by analysing individual attributes and behaviours to provide a more specific understanding of how individual traits contribute to perceptions of good and bad listeners.

Future research can further consider conditions under which listening attributes and behaviours mediate the effects of being perceived as a good or bad listener. For example, research could investigate how different communication contexts, such as high-stress situations versus casual interactions, might alter the extent to which listening attributes or behaviours mediate the effects of being perceived as a good or bad listener. Additionally, research could explore whether the strength of the relationship between listener and speaker moderates the impact of specific listening attributes or behaviours on perceptions, thereby clarifying the conditions under which these factors are most influential in shaping outcomes.

Across our studies, we deliberately focused on listening in a dyadic conversation. We made this decision because it aligns with the most common form of real-world conversation, as well as being consistent with most research that has studied listening. Listening occurs in a myriad of diverse contexts. Much conversation occurs in a group context, which can include examples such as a professor lecturing to hundreds of students to two opposing political groups debating a particular policy topic. What people think constitutes good and bad listeners might differ across such contexts. Similarly, our perceptions of what makes a good or bad listener might also differ depending upon whether we are in a conversation with an in-group or outgroup member, and our mental representations of what makes a good or bad listener might differ in these contexts (see [93]).

A potential limitation of Study 3 lies in the use of 'non-narcissist' as a comparative category, which may introduce variability in participants’ interpretations. While prior research has not explicitly operationalized 'non-narcissist' as a standalone construct, studies examining narcissistic traits have effectively used individuals with lower levels of narcissism as control stimuli [94–96]. These studies illustrate the feasibility of comparing narcissistic and less narcissistic individuals in exploring trait-related perceptions. The potential 'featureless' appearance of the non-narcissist classification image may reflect the broader variability in participants' mental representations of this category, rather than a methodological artefact. To address potential variability, future research could enhance methodological clarity by providing participants with explicit definitions or illustrative examples of the intended non-narcissistic traits.

Of course, listening is only one component of dyadic communication. It is important to consider the attributes and behaviours associated with good versus bad speakers, and how good and bad speakers are visually represented by others. Once again, it is conceivable that the attributes and behaviours we associate with (for example) good speakers might depend upon what we know about the speaker (e.g. do we support the same political party?).

Finally, with the development of artificial intelligence and the use of voice-based Chat Bots, future research might address how people perceive listeners in these emerging, contemporary forms of dyadic interactions. As programs such as ChatGPT become incorporated into therapeutic and medical services [97,98], these programs could seek to further enhance their effectiveness by, for example, potentially designing stimuli that align with users’ representations of a good listener, to help make the individual’s experience more aligned with being the recipient of good listening.

### 9.2. Conclusion

This research advances our understanding of how listeners are perceived in social relationships, revealing that good listeners are consistently viewed as warmer, more competent and more humble than bad listeners. This research is among the first to empirically link listening to personal values, showing that good listeners are associated with higher self-transcendence and openness values. Parallel mediation indicated that the effects of the perception of a good listener were overall, simultaneously mediated by good and bad attributes and good behaviours but not by bad behaviours.

## Data Availability

This manuscript adheres to transparency and openness guidelines (Appelbaum et al., 2018). The data, codes and preregistrations (Studies 2 and 3) are available at this OSF link: [99]. Supplementary material is available online [100].

## Footnotes

^1^
For a separate project, Study 1 participants completed a measure of their values (modelled after the Schwartz Values Scale, see [45]) and rated themselves on a set of attributes (e.g. warm, competent, friendly, capable; 1 = *not at all; 7 = very much*). In all three studies, we also included an exploratory single item asking whether they thought of themselves as good listeners (1 = not at all; 7 = very much).

^2^
Two respondents included age values of 0 and 226. We excluded these when deriving the sample’s mean age.

## References

[1] Hargie O. 2021 Skilled interpersonal communication: research, theory and practice. Abingdon, UK: Routledge. (10.4324/9781003182269)

[2] Itzchakov G, Kluger AN, Castro DR. 2017 I am aware of my inconsistencies but can tolerate them. Personal. Soc. Psychol. Bull. **43**, 105–120. (10.1177/0146167216675339) 27856728

[3] Kriz TD, Jolly PM, Shoss MK. 2021 Coping with organizational layoffs: managers’ increased active listening reduces job insecurity via perceived situational control. J. Occup. Health Psychol. **26**, 448–458. (10.1037/ocp0000295) 34351189

[4] Itzchakov G, Weinstein N, Saluk D, Amar M. 2023 Connection heals wounds: feeling listened to reduces speakers’ loneliness following a social rejection disclosure. Personal. Soc. Psychol. Bull. **49**, 1273–1294. (10.1177/01461672221100369) PMC10320710 35726696

[5] Itzchakov G, Weinstein N, Leary M, Saluk D, Amar M. 2024 Listening to understand: the role of high-quality listening on speakers’ attitude depolarization during disagreements. J. Personal. Soc. Psychol. **126**, 213–239. (10.1037/pspa0000366) 37917499

[6] Castro DR, Kluger AN, Itzchakov G. 2016 Does avoidance‐attachment style attenuate the benefits of being listened to? Eur. J. Soc. Psychol. **46**, 762–775. (10.1002/ejsp.2185)

[7] Itzchakov G, Weinstein N. 2021 High-quality listening supports speakers’ autonomy and self-esteem when discussing prejudice. Hum. Commun. Res. **47**, 248–283. (10.1093/hcr/hqab003)

[8] Weinstein N, Huo A, Itzchakov G. 2021 Parental listening when adolescents self-disclose: a preregistered experimental study. J. Exp. Child Psychol. **209**, 105178. (10.1016/j.jecp.2021.105178) 34087604

[9] Weinstein N, Itzchakov G. 2025 Empathic listening satisfies speakers’ psychological needs and well-being, but doesn’t directly deepen solitude experiences: a registered report. J. Exp. Soc. Psychol. **117**, 104716. (10.1016/j.jesp.2024.104716)

[10] Pasupathi M, Rich B. 2005 Inattentive listening undermines self‐verification in personal storytelling. J. Personal. **73**, 1051–1086. (10.1111/j.1467-6494.2005.00338.x) 15958144

[11] Pasupathi M, Hoyt T. 2010 Silence and the shaping of memory: how distracted listeners affect speakers’ subsequent recall of a computer game experience. Memory **18**, 159–169. (10.1080/09658210902992917) 20391180

[12] Castro DR, Anseel F, Kluger AN, Lloyd KJ, Turjeman-Levi Y. 2018 Mere listening effect on creativity and the mediating role of psychological safety. Psychol. Aesthet. Creat. Arts **12**, 489–502. (10.1037/aca0000177)

[13] Bodie GD. 2023 Listening as a positive communication process. Curr. Opin. Psychol. **53**, 101681. (10.1016/j.copsyc.2023.101681) 37625310

[14] Reis HT, Itzchakov G. 2023 'Do you hear me?': understanding the interplay of listening and perceived partner responsiveness. Curr. Opin. Psychol. **52**, 101615. (10.1016/j.copsyc.2023.101615) 37392504

[15] Bodie GD, St. Cyr K, Pence M, Rold M, Honeycutt J. 2012 Listening competence in initial interactions I: distinguishing between what listening is and what listeners do. Int. J. List. **26**, 1–28. (10.1080/10904018.2012.639645)

[16] Itzchakov G, Reis HT, Weinstein N. 2022 How to foster perceived partner responsiveness: high‐quality listening is key. Soc. Personal. Psychol. Compass **16**, e12648. (10.1111/spc3.12648)

[17] Moin T *et al*. 2024 The effects of listening on speaker and listener while talking about character strengths: an open science school-wide collaboration. R. Soc. Open Sci. **11**, 221342. (10.1098/rsos.221342) 39698154 PMC11651903

[18] Kluger AN, Lehmann M, Aguinis H, Itzchakov G, Gordoni G, Zyberaj J, Bakaç C. 2023 A meta-analytic systematic review and theory of the effects of perceived listening on work outcomes. J. Bus. Psychol. **39**, 295–344. (10.1007/s10869-023-09897-5)

[19] Lemay EP, Le BM, Clark MS. 2023 Listening and the pursuit of communal relationships. Curr. Opin. Psychol. **52**, 101611. (10.1016/j.copsyc.2023.101611) 37354571

[20] Sprecher S. 2023 Listening and responsiveness in getting-acquainted processes. Curr. Opin. Psychol. **52**, 101645. (10.1016/j.copsyc.2023.101645) 37399779

[21] Kluger AN, Itzchakov G. 2022 The power of listening at work. Annu. Rev. Organ. Psychol. Organ. Behav. **9**, 121–146. (10.1146/annurev-orgpsych-012420-091013)

[22] Zhou J, Fredrickson BL. 2023 Listen to resonate: better listening as a gateway to interpersonal positivity resonance through enhanced sensory connection and perceived safety. Curr. Opin. Psychol. **53**, 101669. (10.1016/j.copsyc.2023.101669) 37619451

[23] Kluger AN, Mizrahi M. 2023 Defining listening: can we get rid of the adjectives? Curr. Opin. Psychol. **52**, 101639. (10.1016/j.copsyc.2023.101639) 37437381

[24] Kluger AN *et al*. 2021 Dyadic listening in teams: social relations model. Appl. Psychol. **70**, 1045–1099. (10.1111/apps.12263)

[25] Malloy TE, Kluger AN, Martin J, Pery S. 2023 Women listening to women at zero-acquaintance: interpersonal befriending at the individual and dyadic levels. Int. J. List. 1-15. **37**, 2021. (10.1080/10904018.2021.1884080)

[26] Nichols RG. 1948 Factors in listening comprehension. Speech Monogr **15**, 154–163. (10.1080/03637754809374953)

[27] Nichols RG. 1957 Listening is a 10-part skill. Nation’s Bus. **45**, 1.

[28] Nichols RG. 1959 The art of listening. Res. Manag. **2**, 97–117.

[29] Nichols RG. 1962 Listening is good business. Manag. Pers. Q. **1**, 2–9.

[30] Fiske ST. 2018 Stereotype content: warmth and competence endure. Curr. Dir. Psychol. Sci. **27**, 67–73. (10.1177/0963721417738825) 29755213 PMC5945217

[31] Maio GR. 2016 The psychology of human values. Abingdon, UK: Routledge.

[32] Aggarwal P, Castleberry SB, Ridnour R, Shepherd CD. 2005 Salesperson empathy and listening: impact on relationship outcomes. J. Mark. Theory Pract. **13**, 16–31. (10.1080/10696679.2005.11658547)

[33] Drollinger T, B. Comer L. 2013 Salesperson’s listening ability as an antecedent to relationship selling. J. Bus. Ind. Mark. **28**, 50–59. (10.1108/08858621311285714)

[34] Gilbert DA. 2004 Coordination in nurses’ listening activities and communication about patient–nurse relationships. Res. Nurs. Health **27**, 447–457. (10.1002/nur.20043) 15514958

[35] Huang K, Yeomans M, Brooks AW, Minson J, Gino F. 2017 It doesn’t hurt to ask: question-asking increases liking. J. Personal. Soc. Psychol. **113**, 430–452. (10.1037/pspi0000097) 28447835

[36] Bodie GD, Pence ME, Rold M, Chapman MD, Lejune J, Anzalone L. 2015 Listening competence in initial interactions II: applying trait centrality to discover the relative placement of listening competence among implicit competency theories. Commun. Stud. **66**, 528–548. (10.1080/10510974.2015.1039657)

[37] Cuddy AJC, Fiske ST, Glick P. 2008 Warmth and competence as universal dimensions of social perception: the stereotype content model and the BIAS map. In Advances in experimental social psychology, pp. 61–149. London, UK: Elsevier. (10.1016/s0065-2601(07)00002-0)

[38] Kluger AN, Zaidel K. 2013 Are listeners perceived as leaders? Int. J. List. **27**, 73–84. (10.1080/10904018.2013.754283)

[39] Van Quaquebeke N, Gerpott FH. 2023 Tell-and-sell or ask-and-listen: a self-concept perspective on why it needs leadership communication flexibility to engage subordinates at work. Curr. Opin. Psychol. **53**, 101666. (10.1016/j.copsyc.2023.101666) 37597428

[40] Rubin RS, Bartels LK, Bommer WH. 2002 Are leaders smarter or do they just seem that way? Exploring perceived intellectual competence and leadership emergence. Soc. Behav. Personal. **30**, 105–118. (10.2224/sbp.2002.30.2.105)

[41] Bechler C, Johnson SD. 1995 Leadership and listening. Small Group Res. **26**, 77–85. (10.1177/1046496495261004)

[42] Johnson SD, Bechler C. 1998 Examining the relationship between listening effectiveness and leadership emergence. Small Group Res. **29**, 452–471. (10.1177/1046496498294003)

[43] Arieli S, Sagiv L, Roccas S. 2020 Values at work: the impact of personal values in organisations. Appl. Psychol. **69**, 230–275. (10.1111/apps.12181)

[44] Schwartz SH, Bilsky W. 1987 Toward a universal psychological structure of human values. J. Personal. Soc. Psychol. **53**, 550–562. (10.1037//0022-3514.53.3.550)

[45] Schwartz SH. 1992 Universals in the content and structure of values: theoretical advances and empirical tests in 20 countries. In Advances in experimental social psychology advances in experimental social psychology volume 25, pp. 1–65. London, UK: Elsevier. (10.1016/s0065-2601(08)60281-6)

[46] Adler MJ. 1997 How to speak how to listen. London, UK: Simon and Schuster.

[47] Cojuharenco I, Karelaia N. 2020 When leaders ask questions: can humility premiums buffer the effects of competence penalties? Organ. Behav. Hum. Decis. Process. **156**, 113–134. (10.1016/j.obhdp.2019.12.001)

[48] Van Quaquebeke N, Felps W. 2018 Respectful inquiry: a motivational account of leading through asking questions and listening. Acad. Manag. Rev. **43**, 5–27. (10.5465/amr.2014.0537)

[49] Imhof M. 2002 In the eye of the beholder: children’s perception of good and poor listening behavior. Int. J. List. **16**, 40–56. (10.1080/10904018.2002.10499048)

[50] Rogers CR. 1980 A way of being. Boston, USA: Houghton Mifflin.

[51] Carr HL, Vignoles VL. 2011 Keeping up with the Joneses: status projection as symbolic self‐completion. Eur. J. Soc. Psychol. **41**, 518–527. (10.1002/ejsp.812)

[52] Vignoles VL, Regalia C, Manzi C, Golledge J, Scabini E. 2006 Beyond self-esteem: influence of multiple motives on identity construction. J. Personal. Soc. Psychol. **90**, 308–333. (10.1037/0022-3514.90.2.308) 16536653

[53] Kluger AN, Bouskila‐Yam O. 2018 Facilitating listening scale (FLS). In The sourcebook of listening research: methodology and measures (eds DL Worthington, GD Bodie), pp. 272–280. Hoboken, USA: John Wiley & Sons. (10.1002/9781119102991.ch25)

[54] Dotsch R, Todorov A. 2012 Reverse correlating social face perception. Soc. Psychol. Personal. Sci. **3**, 562–571. (10.1177/1948550611430272)

[55] Appelbaum M, Cooper H, Kline RB, Mayo-Wilson E, Nezu AM, Rao SM. 2018 Journal article reporting standards for quantitative research in psychology: the APA publications and communications board task force report. Am. Psychol. **73**, 3–25. (10.1037/amp0000191) 29345484

[56] Faul F, Erdfelder E, Lang AG, Buchner A. 2007 GPower 3: a flexible statistical power analysis program for the social, behavioral, and biomedical sciences. Behav. Res. Methods **39**, 175–191. (10.3758/bf03193146) 17695343

[57] Han R, Proulx T, van Harreveld F, Haddock G. 2023 How people perceive dispositionally (non-) ambivalent others and why it matters. J. Exp. Soc. Psychol. **109**, 104518. (10.1016/j.jesp.2023.104518)

[58] Magazin E, Haddock G, Proulx T. 2024 Progressives derogate in-group members who hold divergent views. Manuscript Submitted for Publication.

[59] Schwartz SH *et al*. Refining the theory of basic individual values. J. Pers. Soc. Psychol. **103**, 663–688. (10.1037/a0029393) 22823292

[60] Bodie GD. 2011 The active-empathic listening scale (AELS): conceptualization and evidence of validity within the interpersonal domain. Commun. Q. **59**, 277–295. (10.1080/01463373.2011.583495)

[61] Elo S, Kyngäs H. 2008 The qualitative content analysis process. J. Adv. Nurs. **62**, 107–115. (10.1111/j.1365-2648.2007.04569.x) 18352969

[62] Krippendorff K. 2018 Content analysis: an introduction to its methodology. London, UK: Sage publications. (10.4135/9781071878781)

[63] Hayes AF. 2017 Introduction to mediation, moderation, and conditional process analysis: a regression-based approach. New York, USA: Guilford publications.

[64] Benjamini Y, Hochberg Y. 1995 Controlling the false discovery rate: a practical and powerful approach to multiple testing. J. R. Stat. Soc. **57**, 289–300. (10.1111/j.2517-6161.1995.tb02031.x)

[65] Benjamini Y, Hochberg Y. 2000 On the adaptive control of the false discovery rate in multiple testing with independent statistics. J. Educ. Behav. Stat. **25**, 60–83. (10.3102/10769986025001060)

[66] Narum SR. 2006 Beyond Bonferroni: less conservative analyses for conservation genetics. Conserv. Genet. **7**, 783-787 811–811. (10.1007/s10592-006-9189-7)

[67] Brown-Iannuzzi JL, Dotsch R, Cooley E, Payne BK. 2017 The relationship between mental representations of welfare recipients and attitudes toward welfare. Psychol. Sci. **28**, 92–103. (10.1177/0956797616674999) 27879320

[68] Haddock G, Foad CMG, Thorne S. 2022 How do people conceptualize mindfulness? R. Soc. Open Sci. **9**, 211366. (10.1098/rsos.211366) 35345432 PMC8941388

[69] Hanel PHP, Roy D, Taylor S, Franjieh M, Heffer C, Tanesini A, Maio GR. 2023 Using self-affirmation to increase intellectual humility in debate. R. Soc. Open Sci. **10**, 220958. (10.1098/rsos.220958) 36756062 PMC9890103

[70] Itzchakov G, Reis HT, Rios K. 2024 Perceiving others as responsive lessens prejudice: the mediating roles of intellectual humility and attitude ambivalence. J. Exp. Soc. Psychol. **110**, 104554. (10.1016/j.jesp.2023.104554)

[71] Porter T, Schumann K. 2018 Intellectual humility and openness to the opposing view. Self Identity **17**, 139–162. (10.1080/15298868.2017.1361861)

[72] Lehmann M, Kluger AN, Van Tongeren DR. 2023 Am I arrogant? Listen to me and we will both become more humble. J. Posit. Psychol. **18**, 350–362. (10.1080/17439760.2021.2006761)

[73] Dotsch R. 2016 rcicr: reverse correlation image classification toolbox. R package version 0.3, 4.

[74] Smith SJ, Proulx T, Haddock G. 2024 What narcissists look like and why it’s important 10.1177/01461672251339014 PMC13310266 40400459

[75] Cone J, Brown-Iannuzzi JL, Lei R, Dotsch R. 2020 Type I error is inflated in the two-phase reverse correlation procedure. Soc. Psychol. Personal. Sci. **12**, 760–768. (10.1177/1948550620938616)

[76] Camp NP, Voigt R, Jurafsky D, Eberhardt JL. 2021 The thin blue waveform: racial disparities in officer prosody undermine institutional trust in the police. J. Personal. Soc. Psychol. **121**, 1157–1171. (10.1037/pspa0000270) 34264731

[77] Oliver A, Tracy RE, Young SG, Wout DA. 2024 Black + white = prototypically black: visualizing black and white people’s mental representations of black–white biracial people. Personal. Soc. Psychol. Bull. **50**, 1113-1127 014616722311640. (10.1177/01461672231164026) 37052339

[78] Rougier M, De Houwer J. 2023 Unconstraining evaluative conditioning research by using the reverse correlation task. Soc. Psychol. Personal. Sci. 19485506231217526. (10.1177/19485506231217526)

[79] Rougier M, Schmitz M, Nuel I, Fayant MP, Subra B, Alexopoulos T, Yzerbyt V. 2025 It is not only whether I approach but also why i approach: a registered report on the role of action framing in approach/avoidance training effects. J. Exp. Soc. Psychol. **117**, 104697. (10.1016/j.jesp.2024.104697)

[80] Owens BP, Johnson MD, Mitchell TR. 2013 Expressed humility in organizations: implications for performance, teams, and leadership. Organ. Sci. **24**, 1517–1538. (10.1287/orsc.1120.0795)

[81] Brown-Iannuzzi JL, McKee S, Gervais WM. 2018 Atheist horns and religious halos: mental representations of atheists and theists. J. Exp. Psychol. **147**, 292–297. (10.1037/xge0000376) 29154618

[82] Barnett MD, Sharp KJ. 2017 Narcissism, gender, and evolutionary theory: the role of private and public self-absorption. Personal. Individ. Differ. **104**, 326–332. (10.1016/j.paid.2016.08.008)

[83] Rubinstein G. 2017 Narcissistic leadership in organizations: a two-edged sword. In Redefining management (eds V Muhlbauer, W Harry), pp. 155–178. London, UK: Springer International Publishing. (10.1007/978-3-319-69209-8_9)

[84] Hayes AF. 2018 Partial, conditional, and moderated moderated mediation: quantification, inference, and interpretation. Commun. Monogr. **85**, 4–40. (10.1080/03637751.2017.1352100)

[85] Lipetz L, Kluger AN, Bodie GD. 2020 Listening is listening is listening: employees’ perception of listening as a holistic phenomenon. Int. J. List. **34**, 71–96. (10.1080/10904018.2018.1497489)

[86] Elfenbein HA. 2013 Nonverbal dialects and accents in facial expressions of emotion. Emot. Rev. **5**, 90–96. (10.1177/1754073912451332)

[87] Sutherland CAM, Young AW. 2022 Understanding trait impressions from faces. Br. J. Psychol. **113**, 1056–1078. (10.1111/bjop.12583) 35880691 PMC9796653

[88] Hanel PHP, Vione KC. 2016 Do Student Samples Provide an Accurate Estimate of the General Public? PLoS ONE **11**, e0168354. (10.1371/journal.pone.0168354) 28002494 PMC5176168

[89] Arnett JJ. 2016 The neglected 95%: why American psychology needs to become less American. Washington, USA: American Psychological Association. (10.1037/14805-008) 18855491

[90] Henrich J, Heine SJ, Norenzayan A. 2010 The weirdest people in the world? Behav. Brain Sci. **33**, 61–83. (10.1017/s0140525x0999152x) 20550733

[91] Kiewitz C, Weaver JB, Brosius HB, Weimann G. 1997 Cultural differences in listening style preferences: a comparison of young adults in Germany, Israel, and the United States. Int. J. Public Opin. Res. **9**, 233–247. (10.1093/ijpor/9.3.233)

[92] Roebuck DB, Bell RL, Raina R, Lee CE). 2016 Comparing perceived listening behavior differences between managers and nonmanagers living in the United States, India, and Malaysia. Int. J. Bus. Commun **53**, 485–518. (10.1177/2329488415572789)

[93] Proulx T, Costin V, Magazin E, Zarzeczna N, Haddock G. 2023 The progressive values scale: assessing the ideological schism on the left. Personal. Soc. Psychol. Bull. **49**, 1248–1272. (10.1177/01461672221097529) 35678013

[94] Holtzman NS. 2011 Facing a psychopath: detecting the dark triad from emotionally-neutral faces, using prototypes from the Personality Faceaurus. J. Res. Personal. **45**, 648–654. (10.1016/j.jrp.2011.09.002)

[95] Holtzman NS, Strube MJ. 2013 People with dark personalities tend to create a physically attractive veneer. Soc. Psychol. Personal. Sci. **4**, 461–467. (10.1177/1948550612461284)

[96] Medlin MM, Sacco DF, Brown M. 2020 The relation between narcissistic personality traits and accurate identification of, and preference for, facially communicated narcissism. Evol. Psychol. Sci. **6**, 166–173. (10.1007/s40806-019-00224-x)

[97] Garg RK, Urs VL, Agrawal AA, Chaudhary SK, Paliwal V, Kar SK. 2023 Exploring the role of ChatGPT in patient care (diagnosis and treatment) and medical research: a systematic review. Health Promot. Perspect. **13**, 183–191. (10.34172/hpp.2023.22) 37808939 PMC10558973

[98] Javaid M, Haleem A, Singh RP. 2023 ChatGPT for healthcare services: an emerging stage for an innovative perspective. BenchCouncil Trans. Benchmarks Stand. Eval. **3**, 100105. (10.1016/j.tbench.2023.100105)

[99] Anonymous Contributors. 2025 Listening reverse correlation https://osf.io/rz8p6/?view_only=a519d77551d24e49ae78f571aa15579a

[100] Itzchakov G, Haddock G, Smith S. 2025 Supplementary material from: How Do People Perceive Listeners? FigShare. (10.6084/m9.figshare.c.7750310) PMC11978453 40206850

